# A novel multicopper oxidase (laccase) from cyanobacteria: Purification, characterization with potential in the decolorization of anthraquinonic dye

**DOI:** 10.1371/journal.pone.0175144

**Published:** 2017-04-06

**Authors:** Sumbul Afreen, Tooba Naz Shamsi, Mohd Affan Baig, Nadeem Ahmad, Sadaf Fatima, M. Irfan Qureshi, Md. Imtaiyaz Hassan, Tasneem Fatma

**Affiliations:** 1 Cyanobacterial Biotechnology laboratory, Department of Biosciences, Jamia Millia Islamia, New Delhi, India; 2 Department of Biotechnology, Jamia Millia Islamia, New Delhi, India; 3 Center for Interdisciplinary Research in Basic Sciences, Jamia Millia Islamia, New Delhi, India; Russian Academy of Medical Sciences, RUSSIAN FEDERATION

## Abstract

A novel extracellular laccase enzyme produced from *Spirulina platensis* CFTRI was purified by ultrafiltration, cold acetone precipitation, anion exchange and size exclusion chromatography with 51.5% recovery and 5.8 purification fold. The purified laccase was a monomeric protein with molecular mass of ~66 kDa that was confirmed by zymogram analysis and peptide mass fingerprinting. The optimum pH and temperature of the enzyme activity was found at 3.0 and 30°C using ABTS as substrate but the enzyme was quite stable at high temperature and alkaline pH. The laccase activity was enhanced by Cu+2, Zn+2 and Mn+2. In addition, the dye decolorization potential of purified laccase was much higher in terms of extent as well as time. The purified laccase decolorized (96%) of anthraquinonic dye Reactive blue- 4 within 4 h and its biodegradation studies was monitored by UV visible spectra, FTIR and HPLC which concluded that cyanobacterial laccase can be efficiently used to decolorize synthetic dye and help in waste water treatment.

## Introduction

Laccases (benezenediol:oxygen oxidoreductase (EC 1.10.3.2) are group of oxidases that contains multi copper atoms and catalyzes the oxidation of different phenolic and non-phenolic compounds with simultaneous reduction of oxygen to water [1]. Laccases are found mostly as monomers, dimmers and are generally extracellular glycoproteins containing four copper atoms per monomer. The active site consists of at least one type-1 (T1) copper, associated with oxidation of substrates; one type-2 (T2) and two type-3 (T3) coppers arranged in a trinuclear cluster, where reduction of molecular oxygen occurs. The low substrate specificity makes this enzyme interesting for commercial biotechnology and environmental applications such as food and cosmetics industries, paper and pulp industries, textile industries, as well as biotransformation of environmental pollutants, biosensor, biofuel and organic synthesis applications [2]. The presence of laccase are extensively reported among plants, fungi and in some bacteria that are involved in diverse physiological functions, lignin degradation, pigmentation, pathogenesis, melanin production and spore coat resistance [3–5]. Usually, fungi are the most efficient laccase producers and commercially been used till date whereas, fewer studies have been conducted in prokaryotes [6]. In cyanobacterial strain, only *Phormidium valderianum* and *Oscillatoris boryana* shown to have laccase activity till date but their purification has not been done so far. Bacterial laccases reported to have much higher thermostability as compared to fungal laccases [4, 7]. Recently, first laccase purification from green algae *Tetracystis aeria* was reported [8]. The vast application of laccases in various biotechnological sector requires high amounts of cost effective enzyme production and hence scientist needs adequate attention towards exploration of efficient laccase producer.

Cyanobacteria are prokaryotic organism that have capabilities to survive under extreme environment. Due to phototrophic mode of nutrition, short generation time and easy mass cultivation as compared to fungal source, cyanobacteria may be used as potential candidate for laccase production. The low production yield and high cost of purification procedures of laccases from native sources are not suitable for large-scale production in industrial purposes. However, heterologous expression favours the high market demands of higher laccase productivity in shorter duration with desired properties such as different substrate specificities, enhanced stabilities and cost effectiveness. It has reported that fungal laccases are glycoproteins with carbohydrate contents between 5 to 30% and glycosylation is important for secretion, activation, structure, and stability of fungal laccases [9]. Glycosylation causes problems in heterologous expression of fungal laccases that cannot be overcome easily and that make expression in prokaryotic systems nearly impossible [10]. However, cyanobacterial laccases can be overproduced more easily in heterologous host like *Escherichia coli* due to development of host-vector system for prokaryotic expression. [11]. Very few studies have been reported on the degradation of textile dyes through cyanobacteria and enzyme involvement in degradation of recalcitrant compounds such as dye and dyestuffs have not yet been characterized [12, 13]. Textile dyes form a large group of organic substances posing undesirable and toxic effects on the environment. Due to complex aromatic structure these dyes are not easily degraded and stable against light, water and oxidizing agents. Once discharged in water bodies, they reduce transparency, thereby affecting the photosynthetic activity and dissolved oxygen concentration for the aquatic life. Thus, there is a need to decolorize textile dye. Various physical and chemical treatments such as precipitation, photo degradation, adsorption and chemical degradation are costly, time-consuming and pose methodological disadvantages [14]. Thus, biological process is considered environmental friendly and effective tool in the decolorization of textile dyes [15]. Considering the potential application of laccase for solving environmental problems, in present study, the purification and characterization of cyanobacterial *Spirulina platensis* CFTRI laccase was done and the decolorization of anthraquinonic dye Reactive blue 4 by purified laccase was evaluated.

## Materials and methods

### Chemicals

2, 2’ Azino bis[3 ethylbenzthiazoline 6 sulfonate] (ABTS), acrylamide, ammonium persulfate, bis-acrylamide, coomassie brilliant blue R-250, and TEMED (N, N, N’, N’- Tetramethyl ethylenediamine), Sephadex G-100, Reactive Blue-4 were purchased from Sigma-Aldrich. DEAE cellulose and Guaiacol were purchased from Merck, India. Protein marker was purchased from Genei, Bangalore. All other chemicals used were of analytical grade.

### Microorganism

*Spirulina platensis* CFTRI was obtained from CFTRI, Mysore. The culture was maintained at light intensity 2000 ± 200 lux; photoperiod 12:12 h light: dark phase and temperature 29 ± 1°C. The culture was grown in 500 ml Erlenmeyer flask containing 200 ml Zarrouk’s medium [16].

### Assay for laccase activity

Laccase activity was determined spectrophotometrically by monitoring the oxidation of 2mM 2,2-azino-bis-[3-ethyl benzothiazoline- 6-sulphonic acid] (ABTS) in 0.1 M glycine HCl buffer (pH 2.0) at 420 nm for 1 min with few modification [17]. The assay mixture contained 100 μl of culture filtrate, 100 μl of ABTS substrate and 800 μl of glycine HCl buffer and was assayed at room temperature. The blank contain the entire constituent except the active enzyme. Laccase was expressed as enzyme unit per litre i.e. (μmol min-1) L-1

### Laccase purification

The culture was grown in 4 different 1L Erlenmeyer flask containing 500 ml Zarrouk’s medium in each flask at 30°C. The culture was then induced with (1mM 2,5 Xylidine + 100μm guaiacol) at the time of inoculation. Based on the time course study (data not shown), the laccase activity reached maximum on 4^th^day. Hence, the culture was taken and centrifuged at 8000g for 20 min. The pellet was discarded and the supernatant (culture filtrate) was used for the purification steps. The culture filtrate (2000 ml) was concentrated to 180 ml by ultrafiltration cell using Amicon 8200, YM-30 membrane through a membrane filter (molecular weight cut off 10 kDa) [18] and the proteins were salted out with cold acetone. For this the supernatant were incubated with 4 volume of chilled cold acetone and was incubated for 90 min at -20oC. Centrifugation was done for 30 mins at 10,000×g to collect the protein precipitate which was dissolved in minimal amount of 50mM Tris HCl buffer (pH 8.0) and then extensively dialyzed against same buffer for 24 h. The protein was then concentrated through ultrafiltration and loaded onto anion exchange column (DEAE cellulose) that had been pre equilibrated with Tris HCl buffer (50 mM, pH 8.0). The washing of unbound proteins was done with Tris HCl buffer (50 mM; pH 8.0) and the elution was done by using increasing concentration of NaCl (0–1 M). Fraction collector (LKB Broma 7000 ultra fraction collector) was used to collect 2ml of fractions, whose protein content was determined by measuring the absorbance at 280 nm. The laccase activity of each fraction was determined by the same method as discussed earlier. The laccase rich fractions were pooled together, concentrated, dialyzed, and further purified using sephadex G-100 column. Elution was done similarly as in case of anion exchange chromatography and the purified protein was concentrated, filtered through 0.22 μm and stored at −20°C, until further use.

### Protein estimation

Protein concentration was measured by Lowry method [19]. BSA (1 mgml^-1^) was used as a standard and the concentration was expressed in milligram per milliliter (mgml^-1^).

### Characterization of purified laccase

#### SDS PAGE analysis

Sodium dodecyl sulfate-polyacrylamide gel electrophoresis (SDS-PAGE) was carried out on 12.5% polyacrylamide slab gel by Laemmli method [20]. The molecular mass of purified laccase was determined using medium range (6.3–97 kDa) molecular weight markers.

### Zymogram analysis of laccase

Activity staining of laccases was performed after denaturing (or native gel electrophoresis). After completion of the electrophoresis, the gel was allowed to stand in 100ml of Glycine (100mM, pH-2.0) containing 2mM ABTS 30 minutes in dark. Laccase activity bands were confirmed by the development of green coloured bands. Laccase activity was also visualized in the gel using 1 mM guaiacol dissolved in sodium citrate buffer (pH 4.5) as substrate. After approximately 5 to 20 minutes, a yellowish-brown colour appeared at the position of the laccase.

### In- gel tryptic digestion and MALDI-TOF MS/MS for protein identification

The protein band from SDS gel was cut and picked in 0.2 ml microcentrifuge tube. Trypsin digestion of excised protein was done by the method of Bagheri et al. [21]. The peptide mass fingerprinting was performed on a MALDI-TOF-TOF MS analyzer (ABSCIEX TOF/TOF 5800, Applied Biosystems, USA) and the protein identification (ID) were obtained using result-dependent analysis (RDA) by ProteinPilot™ software (Version 3.2, USA). The resulting peptide mass finger-printings (PMFs) were used to identify both proteins by searching the SWISS-PROT and NCBI-nr databases using the Mascot 2.0 search engine with fragment mass tolerance of ±0.3 Da.

### Catalytic property of laccase with respect to physiochemical conditions

#### Effect of pH on activity and stability of purified laccase

The optimum pH of the purified laccase was determined with ABTS substrate prepared in varying pH (2.0–11.0) at 30°C. The buffer systems used were 100 mM ranging from KCl- HCl (1.0–1.5), Glycine-HCl (2.0–3.5), Na- acetate (4.0–5.5), Phosphate buffer (6.0–7.5), Tris HCl (8.0–9.0 and Glycine-NaOH (9.5–11.0). For pH stability, purified laccase was pre- incubated at pH 1.0–11.0 for 1h and the residual activity was determined using ABTS as substrate.

#### Effect of temperature on activity and stability of purified laccase

The optimum temperature for assay of purified laccase was studied by measuring the laccase activity at optimum pH and temperatures from 10°C to 80°C as describe previously. Thermo stability of purified laccase was determined by incubating the enzyme at temperature from 20°C -80°C with the increment of 10°C for different time period for 180 min. and residual activity was determined using ABTS as the substrate.

#### Effect of inhibitor and metal ions on the activity of purified laccase

The effects of several potential inhibitors i.e. sodium azide, L-cystein, thiourea, EDTA, thioglycolic acid and metal ions i.e. cobalt (Co+2), copper (Cu+2), mercury (Hg+2), magnesium (Mg+2), manganese (Mn+2) and zinc (Zn+2) were determined at its optimum pH (3.0) and temperature (30°C). The purified laccase was pre-incubated with inhibitors and metal ion for 15 min before assaying with ABTS and relative activity was measured using ABTS as substrate.

#### Effect of organic solvent

The effects of organic solvents methanol, ethanol, acetonitrile, dimethyl sulfoxide (DMSO) and N, N dimethylformamide (DMF) at 10% were determined its optimum pH (3.0) and temperature (30°C). The purified laccase was pre-incubated with organic solvents for 15 min and relative activity was measured ABTS as substrate.

### Dye decolorization and biodegradation studies of anthraquinonic dye

The reaction mixture contain dye solution [Reactive Blue 4 (100 mgl^-1^)], was incubated with purified laccase (1.5 U) in 50 mmolL^-1^ sodium phosphate buffer (4.5) at 30°C. Percent decolorization was checked as per formula

$$D=100\left(A_{\mathrm{ini}}–A_{\mathrm{obs}}\right)/A_{\mathrm{ini}}$$

where D is decolorisation (in %), A_ini_ initial absorbance and A_obs_ observed absorbance

### Extraction of dye decolorized products

The decolorized solution (5ml) obtained after 1 hr of incubation of purified laccase with dye (Reactive Blue 4) and the decolorized product was first extracted with equal amount of ethyl acetate four times and the organic layer was collected Then the organic layer was dried in a rotary evaporator, and dry residues obtained, consisting of decolorized products used for further analysis [22].

### Characterization of decolorized products

#### Visible spectra

The dry residue dissolve in minimal amount of buffer and the absorption spectrum was measured between 200 and 800 nm spectrophotometrically (Spectro UV-VIS-2700, Labomed, INC). The rate of decolorization was determined by the decrease in absorbance under the maximum wavelength of RB-4. The standard dye Reactive Blue 4 was taken as control.

#### Fourier transform infrared spectroscopy (FTIR)

The dry residue was characterized through FTIR using Agilent technology instrument with 32 sample scans and 8 cm^**-1**^ resolution. Different spectra were compared across the 650–4000 cm^**-1**^ region. The standard dye (Reactive Blue 4) was taken as control.

#### High performance liquid chromatography (HPLC)

Each dry residue (test or control sample) was mixed with 2ml of methanol prior to HPLC analysis. The elution of sample was done isocratically using a C18 reversed phase column (RPC -18 phenomenex) and analyzed by using mobile phase consisting of 60% of acetonitryl and 40% water (HPLC grade). The flow rate of mobile phase was 0.5 ml/min, and the UV-VIS detector was set at 285 nm [22].

### Statistical analysis

All experiments were carried out in triplicate and the results were expressed as mean ± standard error/deviation. Two-way ANOVA with Bonferroni post-test using GraphPad Prism Program version 5.00 was performed for Windows, GraphPad Software, San Diego, CA, USA, www.graphpad.com.

## Results and discussion

### Purification (*Spirulina platensis* CFTRI, Mysore)

**Fig 1:**
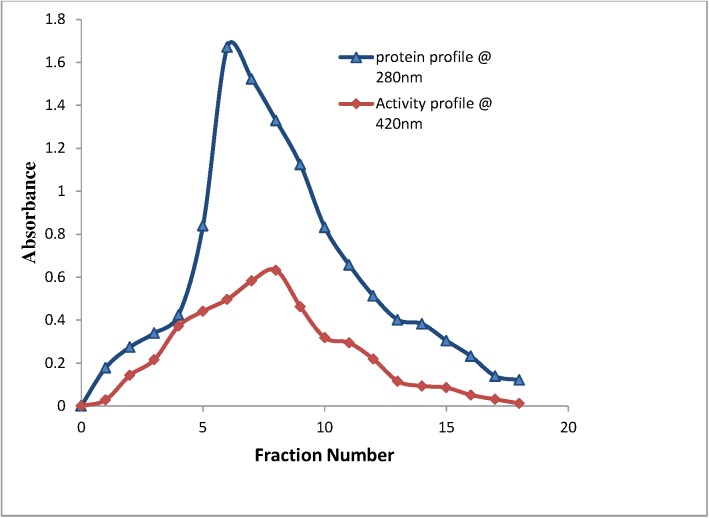
Elution of laccase on DEAE-Cellulose column using 0.2M NaCl. The chromatogram represents concentration of laccase eluted (absorbance A280 at 280 nm) on Y axis with amount of eluted fraction (ml) on X axis. Each fraction were collected and measured for laccase activity (absorbance at A420) using ABTS as substrate

*Spirulina platensis* (CFTRI, Mysore) laccase was purified from the culture filtrate (Table 1). In cold acetone precipitation, the specific activity was increased from 293.42 to 983.6 U/mg protein and its yield and purification factor were 88% and 3.3 fold respectively. The precipitate was dialyzed against 50 mM Tris HCl buffer and applied to a DEAE-cellulose column. Linear gradient elution (0.1-1M NaCl) and fractions were analyzed spectrophotometrically with (ABTS as substrate). The peak showing laccase activity was eluted at 0.2 M NaCl in a DEAE column (Fig 1). In DEAE cellulose column chromatography, the specific activity was increased to 1117.18 U/mg protein with 3.8 fold purification factor and 62.8% yield. The fraction containing major laccase activity were pooled and again dialyzed against 50 mM of Tris HCl buffer and loaded on to Sephadex G- 100 column. The protein concentrations and laccase activity were determined and compared at each step of purification (Table 1). The results depicted downhill slope of concentration and total protein with each passing step whereas an increase in specific activity and fold purification. The specific activity of protein increased from 1117.8 to 1721.5 U/mg along with increase in 3.8 to 5.8 fold purification and 51.5% yield at the end of purification. Mostly laccase purification had been done from fungal sources till date which includes, *Phellinus igniarius*, *Pleurotus ostreatus*, *Trametes gibbosa*, *Trametes hirsuta and Trametes versicolor etc* [6] and only one laccase has been purified and characterized in a green alga [8]. Previously, presence of laccase was reported in *Phormidium valderianum* and *Oscillatoria boryana* but there are no reports on the purification of laccase from cyanobacteria [23]. The presence of laccase gene for putative LAC in cyanobacteria was confirmed in *Synechococcus sp*. *RS9917* by sequencing [24]. The most commonly used method for laccase purification is salt elution from an anion-exchange resin due to higher stability at neutral to alkaline pH and its the isoelectric point (pI) of laccase [25]. The extracel1ular cyanobacterial laccase exhibit strong binding with DEAE cellulose column and elution was done at 0.2 M NaCI gradient. Laccases show variable binding and many studies have also reported the elution from 0.1 M to 0.3 M NaCl gradient [26]. Previously laccase purification was reported from fungi *Pleurotus sajor*-*caju* MTCC 141 with 10.71 fold and 3.46% yield while in bacteria 28.46 fold purification of laccase from *Bacillus tequilensis* SN4 with 13.34% yield was reported [27, 28]. However in green algae *Tetracystis aeria* 120 fold purified having very low yield of 2.5% was obtained [8].

**Table 1:** Protein Estimation and activity profile at each step of purification.

Purification Steps	Volume (ml)	Total laccase activity (U)	Total protein	Specific activity	Fold purification	Yield (%)
			(mg)			
				(U/mg)		
**Concentrated culture filtrate**	100	94500.52	322.5	293.42	1	100
**Acetone precipitate**	50	83213.46	84.6	983.6	3.3	88
**DEAE Cellulose**	20	59432.88	53.2	1117.18	3.8	62.8
**Sephadex G-100**	10	48672.14	26.1	1721.5	5.8	51.5

### Characterization

**Fig 2:**
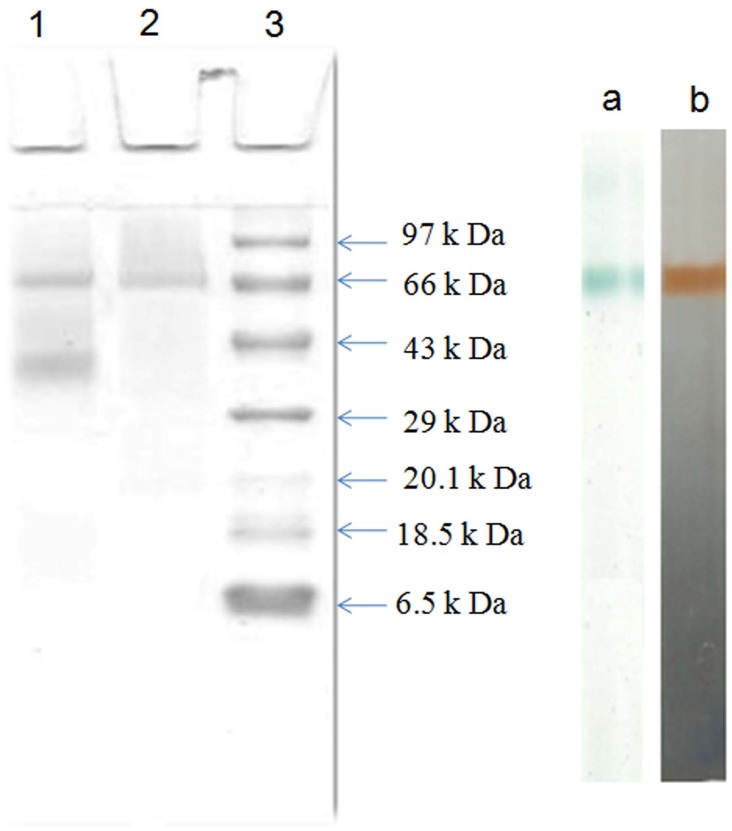
(A) SDS-PAGE of extracellular *Spirulina platensis* CFTRI laccase purified by anion exchange and gel filtration chromatography. Lane 1: DEAE cellulose eluent, Lane 2: Sephadex G-100 eluent, Lane 3: molecular size marker. (B) Zymogram of laccase with (a) ABTS (b) guaiacol

The characterization of purified protein were done through SDS-PAGE followed by its confirmation by zymogram analysis and MALDI-TOF/MS Analysis The purified protein showed a monomeric 66 kDa band on SDS PAGE which was confirmed as laccase on native PAGE during the zymogram analysis (Fig 2A). The green band was developed in presence of substrate ABTS and brown band was developed in presence of substrate guaiacol (Fig 2B). In previous studies many researcher had reported the weight of the fungal and bacterial laccases in the range 50–100 kDa [5, 7]. Activity staining of laccase with ABTS was also reported in fungi *Pischia pastorii* [29] *Pycnoporous sanguineus* [30], *Pleurotus ostreatus* [25], bacteria *Bacillus subtilis* [31] cyanobacteria *Phormidium valderianum* [23] and guaicol staining in *Pleurotus ostreatus* [32], *Pleurotus sajor-caju* [33].

The band excised from SDS-PAGE (Fig 2A) was identified through MALDI TOF-TOF. A band at ~66-kDa was digested with trypsin into 5 fragments in the range of 2070.0339–3214.6296 as shown in the peptide mass fingerprint (Table 2). The Peptide sequence of purified protein from *Spirulina platensis* CFTRI obtained after MALDI-TOF-MS/MS analysis searched against Swissprot database and Mascot database search confirmed that purified enzyme was laccase (raw data given as S1 File) that resemble with laccase of *Trametes versicolor* with highest score of 311 and 5 query match (Matric science, Mascot search result) and 24% protein sequence coverage (S2 File and S3 File.). The presence of laccase in *Bacillus tequilensis* and *Pleurotus ostreatus* HP-1 were also confirmed through MALDI-TOF/MS analysis [25, 28].

**Table 2:** List of peptide fragments obtained after trypsinization (MALDI-TOF/MS-MS analysis).

S. No.	Mass Mr.	Range	Peptide Sequence
1	2070.033	18–59	R.SLAAIGPVASLVVANAPVSPDDFLRDAIVVNGVVPSPLITGK.K
2	2470.076	157–17	R.YDVDNESTVITLTDWYHTAAR.L
3	1514.785	182–196	R.FPLGADATLINGLGR.S
4	1975.9897	281–30	R.ANPNFGTVGFAGGINSAILR.Y
5	3214.6296	301–33	R.YQGAPVAEPTTTQTPSVIPLIETNLHPLAR.M

#### Effect of pH on activity and stability of purified laccase

**Fig 3:**
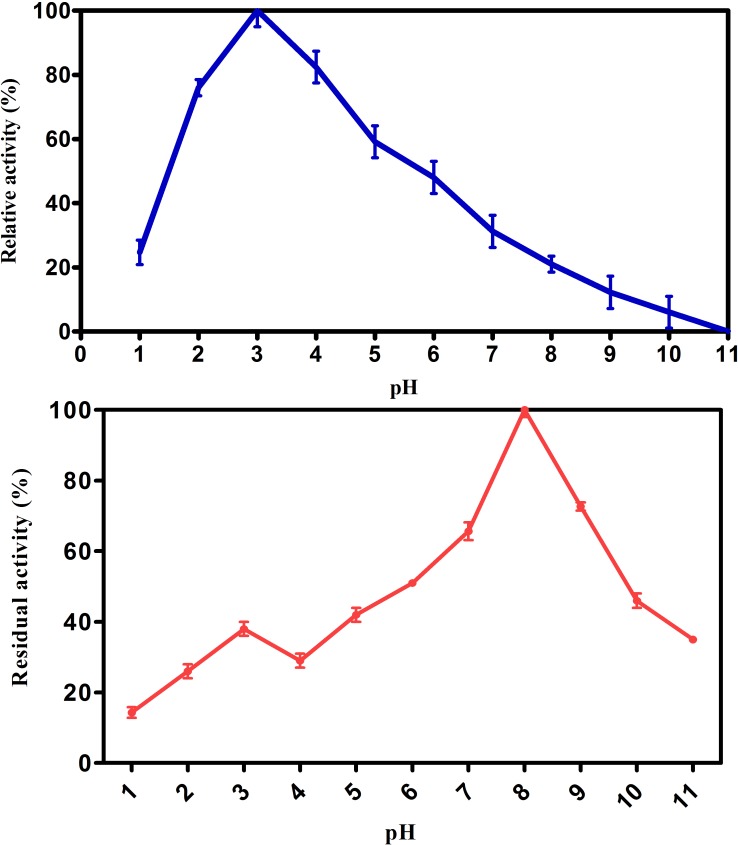
Effect of pH on (a) laccase activity (b) laccase stability. Residual activity was measured using ABTS substrates. Values are mean of triplicates ± S.E

The optimum pH of the purified laccase from *Spirulina platensis* CFTRI, Mysore was ~3.0 with ABTS substrate (Fig 3A). Similar pH was reported for laccase isolated *Pleurotus* sp, *Pycnoporus sanguineus*, *Ganoderma lucidum*, *Pycnoporus* sp [34, 35]. It was completed inactivated at pH-11 which might be due to the binding of a hydroxide anion to the trinuclear coppers of laccase that interrupts the internal electron transfer from T1 to trinuclear centre and ionization of pI [36]. It was reported that the oxidation rate of different substrate gradually decreased at higher pH that might be due to ionization of critical amino acids (Asp and Glu) [37]. The pH stability of laccase was determined and it retained 100% activity after 1 h at ~pH 8.0 and found to be most stable at this pH (Fig 3B). Similar results were obtained with laccase of *Peniophora sp***,** *Cerrena unicolor* strain 137, γ-proteobacterium JB and *Carica papaya* [38–41].

#### Effect of temperature on activity and stability of purified laccase

**Fig 4:**
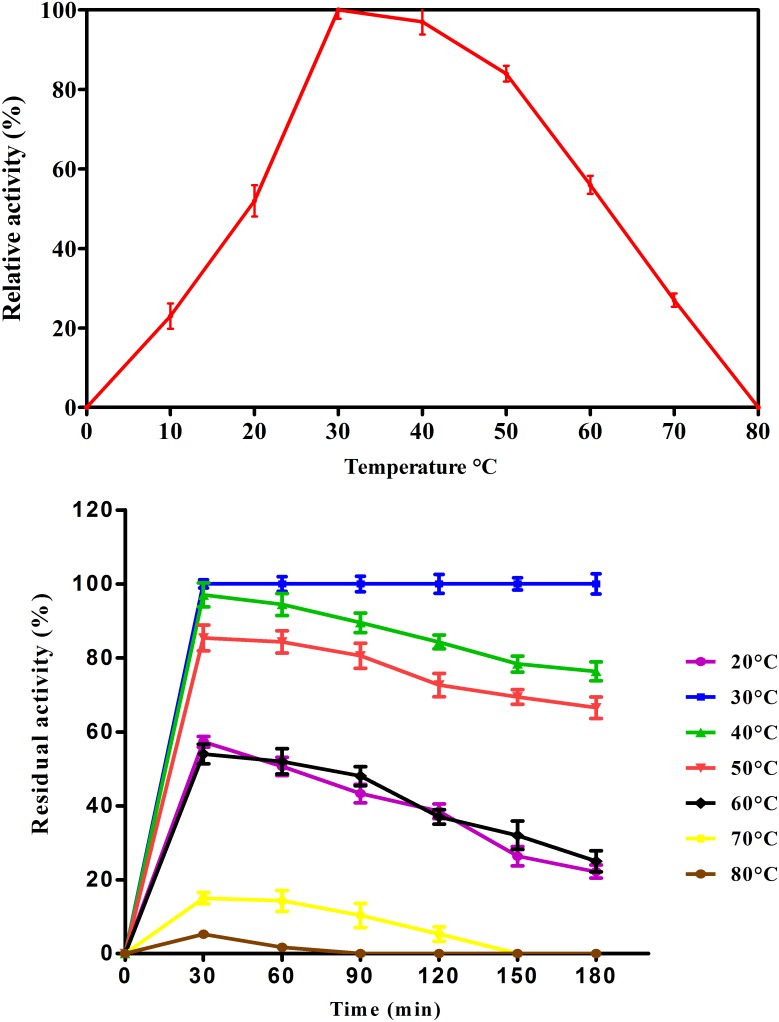
Effect of temperature on (a) laccase activity (b) laccase stability. Residual activity was measured using ABTS substrates. Values are mean of triplicates ± S.E.

Temperature effect showed bell shaped graph. Maximum activity of purified laccase was observed at ~30°C. At 40°C, 97% laccase activity was retained. Further increase in temperature resulted in sharp decrease in enzyme activity which might be due to denaturation of enzyme (Fig 4A). It was reported that laccase from *Pleurotus ostreatus* has showed an optimum temperature at 30°C [42] but found 50°C as optimal temperature in *Pleurotus pulmonarius* and *Pleurotus florida* [43, 44].

Thermal stability study with the purified laccase was conducted at 20°C -80°C for 180 mins and it was found that laccase was quite stable at 30°C-40°C to retain 100% and 97% of its activity after 60 min of incubation. After 30 mins incubation of enzyme at 50°C, 60°C and 70°C, it showed 85.38%, 54.0% and 15.0. 90 mins exposure at 50°C retained 80% activity whereas at 60°C, 70°C and 80°C the residual activity was only 48% and 10%. Increase in temperature resulted in decreased stability of enzyme. After 180 mins incubation at 70°C and 90 mins incubation at 80°C, resulted in zero activity (Fig 4B). The thermal stability of enzymes may be influenced by the presence of hydrophobic or charged residues, which increase enzyme rigidity and restrict conformational changes during substrate binding [45].

#### Effect of inhibitors on laccase activity

**Fig 5:**
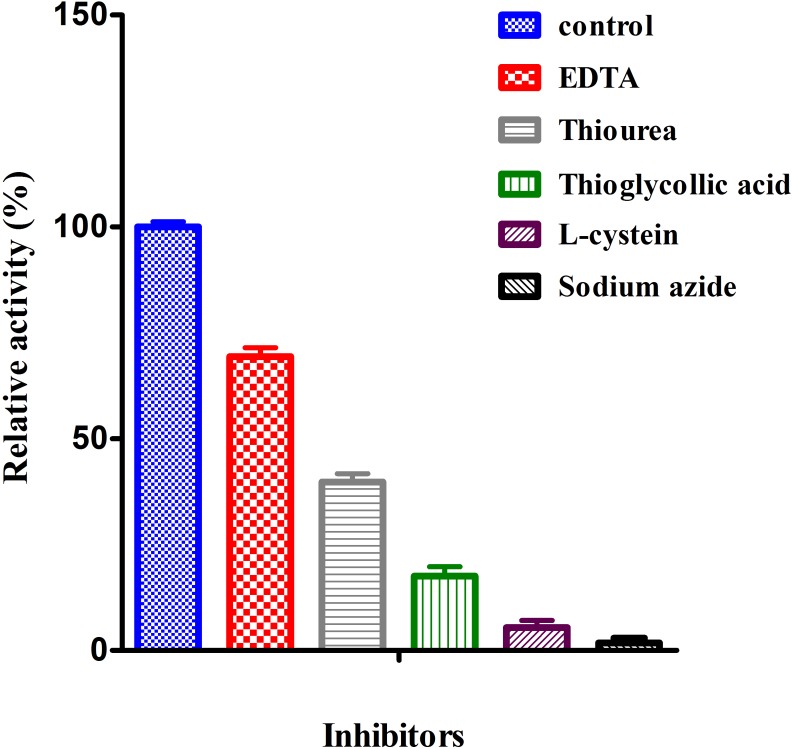
Effect of inhibitors on laccase activity was measured using ABTS as a substrate. Values are mean of triplicates ± S.E

The purified laccase enzyme had been studied presence of studied inhibitors (1mM). The enzyme was inhibited by all studied inhibitors (Fig 5). The order of inhibition was sodium azide (98.23%) > L-cysteine (94.61%) > Thioglycollic acid (82.41%) > Thiourea (60.23%) > EDTA (30.64%). Other scientists have also reported the inhibition of laccase activity by sodium azide *Mannaporthe grisea* [46] and *Pleurotus ostreatus* [25]. Inhibition of laccase activity by thioglycollic acid was reported in *Chaetomium thermophilium* which may be due to effect of copper at the catalytic centre of laccase [47]. It was reported that L-cysteine is one of the effective inhibitor for fungal laccase and like thioglycollic acid, due it effect of copper at the catalytical centre [48]. Many scientistist have reported that EDTA is also an inhibitor of metallo enzymes due to its tendency of forming inactive complexes with inorganic prosthetic cofactors of the enzyme [49]. The laccase activity was inhibited by EDTA was also reported by many researchers [43, 50].

#### Effect of metal ions on laccase activity

**Fig 6:**
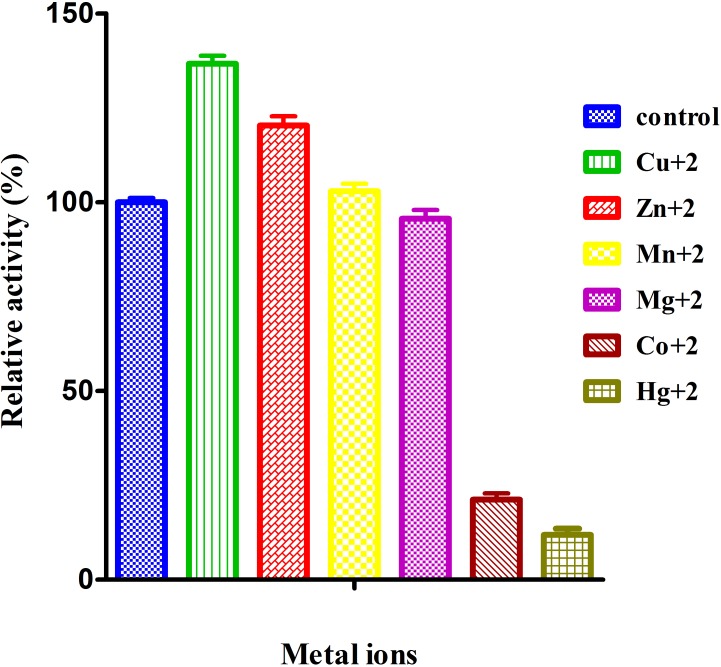
Effect of metal ions on purified laccase activity was measured using ABTS as a substrate. Values are mean of triplicates ± S.E

The effect of metal ions on laccase activity was greatly dependent on its source as well as the type of metals used that strongly affects the catalytic activity of the enzyme [51]. In present study, effect of Cu^**+2**^, Zn^**+2**^, Mn^**+2**^, Hg^**+2**^ and Co^**+2**^ metals was observed for the characterization of laccase. These metals showed two types of behavior. Cu,^**+2**^ Zn^**+2**^ Mn^**+2**^ stimulated the laccase activity while Mg^**+2**^, Co^**+2**^ and Hg^**+2**^ reduced the laccase activity (Fig 6). The order of enhanced laccase activity was Cu^**+2**^ 136.6%) > Zn^**+2**^ (120.31%) > Mn^**+2**^ (102.89%) with respect to control (100%). Other scientist have also reported the stimulatory effect of Cu^**+2**^ and Zn^**+2**^ on laccase activity in *Cladosporium cladosporioides and Streptomyces psammoticus* [52]. The order of reduced laccase activity was Hg^**+2**^(11.89%) > Co^**+2**^ (21.23%) > Mg^**+2**^ (95.60%). Reduction of laccase was reported in *Ganoderma lucidum* [53] and *Bacillus tequilensis* SN4 [28]. Previously it was reported that Hg^**+2**^ ions have strong binding affinity for sulfhydral (- SH) groups which are responsible for the distortion of enzyme structure [54]. In our study Mg^**+2**^ and Co^**+2**^ slightly reduced the laccase activity which might be due to slight unfolding of the laccase in the presence of these metal ions which was contrary to the result on *Bacillus tequilensis* SN4 [28] and on *Trematosphaeria mangrovei* [51] whereas observed no effect of Co^**+2**^ in *Trichoderma harzianum* WL1[49].

#### Effect of organic solvent on laccase activity

**Fig 7:**
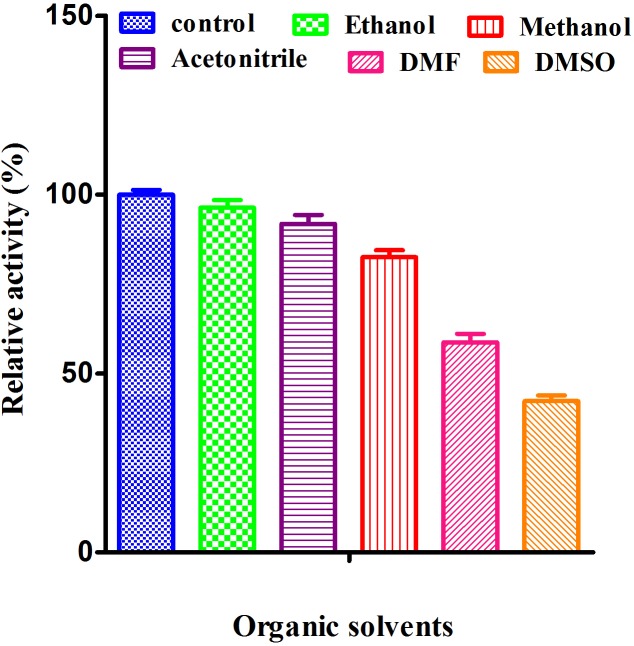
Effect of organic solvents on the activity of purified laccase was measured using ABTS as asubstrate. Values are mean of triplicates ± S.E

In present study, effect of organic solvent like ethanol, acetonitrile, methanol, DMF and DMSO was observed for the characterization of laccase (Fig 7). The order of relative was Ethanol (96.36%) > Acetonitrile (91.80%) > Methanol (82.54%) > DMF (58.76%) > DMSO (42.26%) with respect to control (100%). Similar results were reported in *Cerrena sp*. HYB07 and *Ganoderma fornicatum* [55– 56]. It was also reported that organic solvents inhibit laccase activity by promoting protein unfolding [57]. DMF and DMSO increase relative laccase activity in *Thermobifida fusca* which was contrary to result obtained in our study [58].

#### Dye decolorization

**Fig 8:**
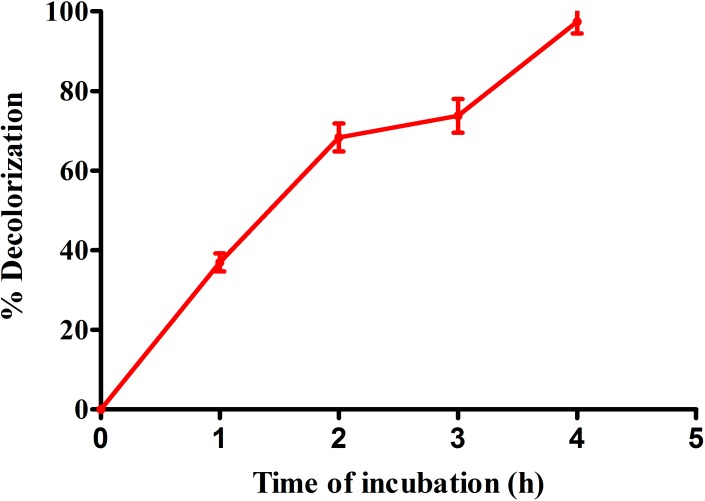
Percent decolorization of Reactive Blue 4 dye by purified laccase of *Spirulina platensis* CFTRI. Values are mean of triplicates + S.E

The purified laccase of *Spirulina platensis* CFTRI, Mysore was more efficiently decolorized anthraquinonic dye RB-4. The dye decolorization potential of purified laccase was much higher in terms of extent as well as time. With crude laccase decolorization RB-4 was 59.36% respectively after 48 h that was enhanced to 97.4% after 4 h with purified laccase (Fig 8). Many scientists have reported that there was no RBBR decolorization by laccase alone, it needs some mediators [59– 60]. It may be concluded that *Spirulina platensis* laccase is much better than other laccase source.

### Biodegradation studies of reactive blue 4

**Fig 9:**
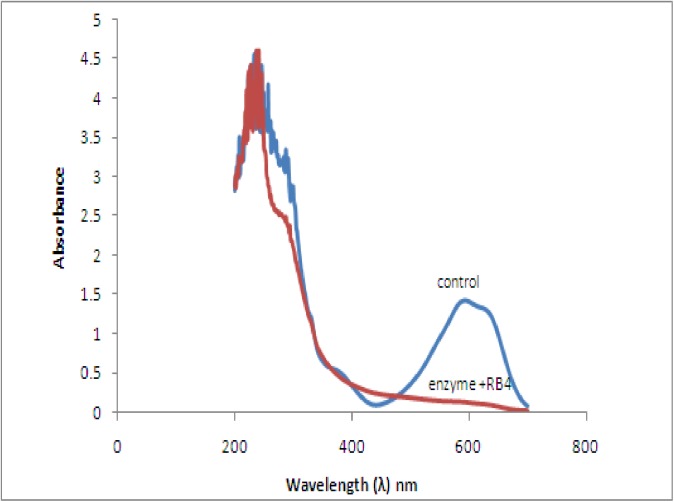
UV- visible spectra of Reactive Blue-4 after 4 h contact with purified laccase (1.5 U) of *Spirulina platensis* CFTRI

The UV absorption spectra of control and anthraquinonic dyes Reactive Blue 4 after 4 h contact with purified laccase of *Spirulina platensis* (CFTRI Mysore) showed almost complete decolorization. The UV-Vis absorbance spectrum of the control RB-4 showed maximum absorbance at wavelengths 595 nm. There was a clear hypsochromic shift observed in UV-Vis absorbance spectrum of the laccase treated Rb-4 (Fig 9). The reduction and shift of the absorbance at the maximum wavelength confirms that RB-4 decolorization was caused by biodegradation [61].

**Fig 10:**
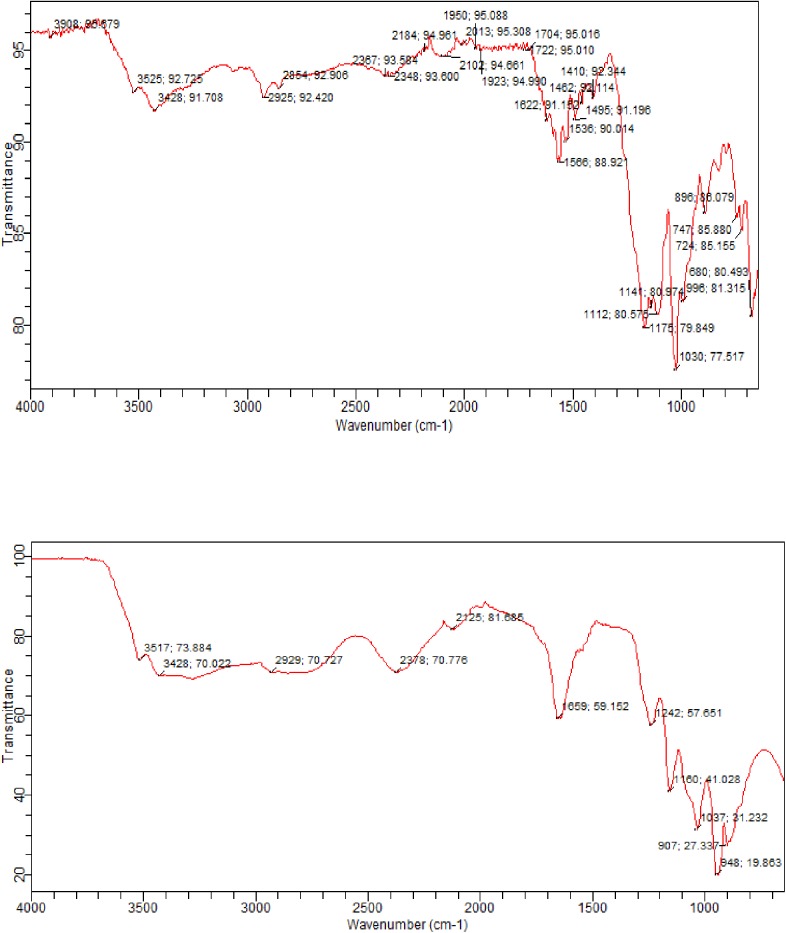
The FTIR spectra (a) Reactive Blue 4 control (b) Laccase treated Reactive Blue 4

The biodegradation of Reactive Blue 4 was monitored using FTIR spectroscopy and a comparison of the transmission spectra before and after decolorization were studied. Fig 10A & 10B) were respectively FTIR spectra of RB4 control, the RB4 sample degraded by purified enzyme. RB- 4 (control) showed a broad peak at 3420.87 cm-1 representing -NH- and hydroxyl (-OH) extensions. The stretching vibrations of C–N band showed the adsorption at 1030, 1118cm-1 and 1175. The peaks at 1566 cm-1 for aromatic ring skeleton cm-1, 1622 cm-1 represent stretching vibration of C = C and 695 cm-1 are -C-S- vibration. The peaks located at 1272.13 cm-1 are those C-O extension. After the laccase treatment, the spectrum of RB4 showed the disappearance of various peaks. At the same time, there were the appearance of new peaks at 1242 cm-1 and 1659 cm-1 corresponding respectively to C-O vibration of acids and C = O deformations of acids and aldehydes that could be due to byproducts which are formed during the Reactive Blue-4 degradation.

**Fig 11:**
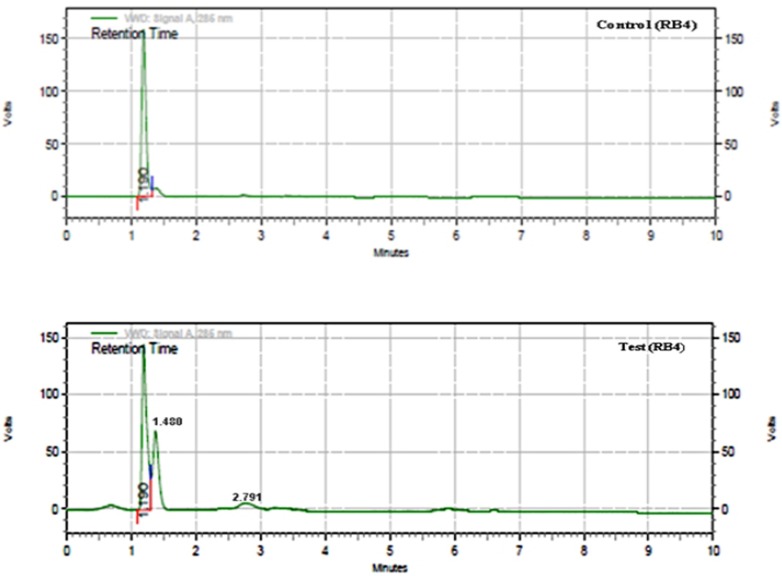
HPLC chromatogram (a) Reactive Blue 4 control (b) Laccase treated Reactive Blue 4

The HPLC analysis of control dye (Reactive blue 4) showed the presence of one major peak at retention time of 1.190 min with concentration of 10.575 mgml-^1^ covering the peak area of 535398649 (Fig 11A). After the dye decolorization process, there was major peak observed at retention time 1.190 but the peak area of dye was decreased 11848018 and the formation of completely different two minor peaks (by products) at retention times of 1.480 mins and 2.79 minswere observed (Fig 11B). The concentration of reactive blue 4 retained after decolorization was 1.106 mgml-^1^ which clearly support the biodegradation of RB4. The HPLC chromatogram of Reactive Blue 4 (control) suggested that similar results were presented during a HPLC follow-up of dye biodegradation [18, 62].

## Conclusion

In summary, a novel extracellular laccase from *Spirulina platensis* CFTRI (Mysore was purified to electrophoretic homogeneity with 51.5% recovery and the purification fold was5.8 after 3 day cultivation. Due to phototrophic mode of nutrition, short generation time and easy mass cultivation, *Spirulina platensis* CFTRI appeared as good candidate for laccase production. The high yield of laccase in short production period are profitable for its industrial application. This study indicates that pure *Spirulina platensis* CFTRI laccase alone could efficiently decolorized anthraquinonic dye Reactive Blue 4 with 4 hwithout any mediators which makes it cost effective & suitable candidate for decolorization of synthetic dyes and help in waste water treatment.

## Supporting information

S1 File Raw data of Maldi-TOF/MS-MS analysis of *Spirulina platensis* CFTRI. (PDF)

S2 File Mascot Search Results of peptides sequences obtained after Maldi-TOF/MS-MS analysis. (PDF)

S3 File A Peptide summary of MALDI-TOF/MS-MS search results that confirms the purified enzyme as laccase. (PDF)

## Abbreviations

2,2’-Azino-bis[3-ethylbenzthiazoline-6-sulfonate]: ABTS
Bovine serum albumin: BSA
Sodium dodecyl sulfate-polyacrylamide gel electrophoresis: SDS-PAGE
(N, N, N’, N’- Tetramethyl ethylenediamine: TEMED
Dimethyl sulfoxide: DMSO
N, N dimethyl formamide: DMF
Reactive Blue 4: RB-4
Fourier Transform Infrared spectroscopy: FTIR
High Performance liquid chromatography: HPLC
