# Supplementary material for: A novel multicopper oxidase (laccase) from cyanobacteria: Purification, characterization with potential in the decolorization of anthraquinonic dye
Source: PLoS One. 2017 Apr 6;12(4):e0175144. doi: 10.1371/journal.pone.0175144 (PMC5383238; doi:10.1371/journal.pone.0175144)
Supplement: S1 File — (PDF) [file pone.0175144.s001.pdf]

```
COM=Project: ABSCIEX SERVICE, Spot Set: ABSCIEX SE
RVICE\080316, Label: B7, Spot Id: 3106644, Peak Li
st Id: 6375246, MS Job Run Id: 57743
832.31317   248226.28
834.31586   25303.055
1243.6581   41444.703
1474.6874   39986.246
1514.7983   125235.51
1515.7916   79041.172
1703.7673   329680.22
1705.7672   45509.906
1974.0721   17910.371
1976.0077   443769.41
1991.9894   673165.25
2059.9446   10208.078
2070.0339   28954.92
2087.9773   459317.09
2150.1462   284842.47
2158.0591   97412.602
2199.0723   7093.0532
2470.0964   112098.69
3214.668    156027.53
3247.3762   8583.7813
BEGIN IONS
PEPMASS=731.45654
CHARGE=1+
TITLE=Label: B7, Spot_Id: 3106644, Peak_List_Id: 63
75342, MSMS Job_Run_Id: 57745, Comment:
158.10397   1983.8264
160.11969   748.33722
162.13521   633.88483
544.27942   265.85394
687.3277    317.37601
688.37122   556.82422
688.61621   336.72806
689.36609   487.13123
690.36945   527.33209
END IONS
BEGIN IONS
PEPMASS=745.47156
CHARGE=1+
TITLE=Label: B7, Spot_Id: 3106644, Peak_List_Id: 63
```

75350, MSMS Job_Run_Id: 57745, Comment:
158.11046   2058.5554
160.12358   481.60345
162.12924   320.21881
174.08832   255.12387
558.27454   434.01587
702.33545   1141.5414
704.35052   1041.4136
END IONS
BEGIN IONS
PEPMASS=751.38513
CHARGE=1+
TITLE=Label: B7, Spot_Id: 3106644, Peak_List_Id: 63
75343, MSMS Job_Run_Id: 57745, Comment:
158.09325   275.51553
159.08986   1162.1959
160.10748   543.31641
162.11975   383.22125
175.11188   1153.7604
250.11571   474.97836
271.15863   484.28418
272.18341   252.85582
278.09842   1648.6415
288.21088   248.05511
440.23602   244.42868
457.23373   855.21265
464.16986   1233.4666
474.25446   205.39075
565.28516   207.6257
566.27063   393.22644
707.27289   230.78896
END IONS
BEGIN IONS
PEPMASS=761.47321
CHARGE=1+
TITLE=Label: B7, Spot_Id: 3106644, Peak_List_Id: 63
75345, MSMS Job_Run_Id: 57745, Comment:
158.08923   2832.5186
160.11298   1460.6461
162.13347   1229.1807
175.11287   409.97318
305.15915   222.28102

```
327.14819   262.33475
532.32111   559.06989
575.01306   256.46585
646.43427   453.47336
716.74054   865.34216
717.38995   827.26801
718.44879   556.8092
719.38525   1058.0598
END IONS
BEGIN IONS
PEPMASS=804.27686
CHARGE=1+
TITLE=Label: B7, Spot_Id: 3106644, Peak_List_Id: 63
75347, MSMS Job_Run_Id: 57745, Comment:
158.10626   2620.0869
160.13342   527.84412
162.15125   337.05261
499.19897   354.01279
604.20068   434.15701
616.25018   1458.4111
618.21637   2102.6609
632.28619   632.00391
634.20929   1105.3838
760.3761    981.83832
762.39453   1369.0787
END IONS
BEGIN IONS
PEPMASS=819.51196
CHARGE=1+
TITLE=Label: B7, Spot_Id: 3106644, Peak_List_Id: 63
75344, MSMS Job_Run_Id: 57745, Comment:
158.09161   2780.8257
160.11255   921.49097
162.13078   863.83881
365.21811   358.69269
439.28024   211.32536
456.25699   201.33397
632.18842   955.39783
649.26929   385.81332
775.383 312.60446
776.60931   903.04846
777.39331   855.3623
```

779.26465   233.17715
END IONS
BEGIN IONS
PEPMASS=832.30383
CHARGE=1+
TITLE=Label: B7, Spot_Id: 3106644, Peak_List_Id: 63
75360, MSMS Job_Run_Id: 57745, Comment:
158.10443   641.92072
527.21545   1871.765
566.34711   968.90961
632.23798   6644.5562
634.81427   1054.292
646.27472   19024.219
648.4668   395.78714
660.30664   1680.9906
661.3205   1431.8704
662.2854   7816.4937
664.39374   517.02203
790.42719   491.62579
END IONS
BEGIN IONS
PEPMASS=1243.645
CHARGE=1+
TITLE=Label: B7, Spot_Id: 3106644, Peak_List_Id: 63
75355, MSMS Job_Run_Id: 57745, Comment:
582.3335   297.59167
625.38757   235.64078
653.37238   361.72614
662.43127   220.17255
800.46063   210.94206
871.38129   266.24579
END IONS
BEGIN IONS
PEPMASS=1474.6757
CHARGE=1+
TITLE=Label: B7, Spot_Id: 3106644, Peak_List_Id: 63
75356, MSMS Job_Run_Id: 57745, Comment:
526.27765   643.88269
626.39874   396.55078
712.39215   1018.7723
763.40826   3041.47
821.43665   437.67667

```
849.46667   1167.418
906.48718   528.91907
949.52838   1215.5913
950.43286   455.00461
1053.4685   432.12885
1086.588    805.26288
END IONS
BEGIN IONS
PEPMASS=1514.7855
CHARGE=1+
TITLE=Label: B7, Spot_Id: 3106644, Peak_List_Id: 63
75357, MSMS Job_Run_Id: 57745, Comment:
112.08976   551.85919
175.12921   1159.8107
402.28223   18174.316
454.27969   650.16461
471.29041   667.48401
499.29587   1443.7933
516.33392   639.86487
517.31567   4632.501
601.31549   778.69012
629.41748   597.28876
742.42938   545.69678
755.39954   483.59512
773.42218   935.2345
858.55072   508.8999
886.46454   692.6488
914.58185   7172.7588
915.52698   4354.8306
1271.6951   592.14197
1471.7673   679.3999
1472.5488   801.07654
END IONS
BEGIN IONS
PEPMASS=1703.7522
CHARGE=1+
TITLE=Label: B7, Spot_Id: 3106644, Peak_List_Id: 63
75362, MSMS Job_Run_Id: 57745, Comment:
111.93231   458.91827
174.9398    3129.9561
304.95184   783.5683
321.96136   797.81396
```

```
324.97134   463.55417
385.92044   552.31525
484.97128   786.34021
515.02069   2776.2117
532.06519   7986.3359
628.99823   2458.3413
646.02478   20785.828
648.13672   855.48639
743.01141   558.03613
744.00507   567.49939
761.04114   979.15045
924.04504   959.80182
942.96924   423.48431
1021.0061   1011.1183
1037.983    482.13962
1659.0619   1345.2003
END IONS
BEGIN IONS
PEPMASS=1742.7133
CHARGE=1+
TITLE=Label: B7, Spot_Id: 3106644, Peak_List_Id: 63
75341, MSMS Job_Run_Id: 57745, Comment:
174.93517   399.49063
532.01428   432.37518
554.00403   205.4893
566.98364   261.30307
571.03802   432.18149
646.10596   396.98468
685.01288   1452.1334
1095.9023   454.78345
1585.8921   731.14557
1697.9595   320.19662
END IONS
BEGIN IONS
PEPMASS=1948.0842
CHARGE=1+
TITLE=Label: B7, Spot_Id: 3106644, Peak_List_Id: 63
75346, MSMS Job_Run_Id: 57745, Comment:
174.9357    294.74512
531.99957   366.79211
646.12335   301.82309
900.12738   221.20755
```

```
1900.1713   200.74107
END IONS
BEGIN IONS
PEPMASS=1975.9897
CHARGE=1+
TITLE=Label: B7, Spot_Id: 3106644, Peak_List_Id: 63
75363, MSMS Job_Run_Id: 57745, Comment:
174.93964   2629.3333
287.9827    465.64255
401.04684   463.09534
472.05234   1223.422
498.93719   646.56891
543.97321   503.62866
559.08917   2561.074
656.04938   1321.8265
673.04822   905.36053
702.03131   591.96362
843.13281   521.30042
900.11804   783.86737
971.1217    692.66846
1175.124    737.60413
1274.2177   536.94269
1432.1804   491.95828
1579.205    641.67517
1790.2284   744.27441
1931.347    698.78058
1932.1599   1149.2212
END IONS
BEGIN IONS
PEPMASS=1991.9727
CHARGE=1+
TITLE=Label: B7, Spot_Id: 3106644, Peak_List_Id: 63
75365, MSMS Job_Run_Id: 57745, Comment:
174.94011   6696.3652
401.05637   1196.8712
472.06152   2519.668
502.96841   1533.0426
559.09796   6056.0059
573.96777   1146.1815
601.9718    1683.0249
656.04657   3163.99
673.04639   1789.809
```

786.0661   1206.2252
787.03851   1408.9847
843.09711   1815.1573
900.117 3205.4883
1205.1532   2206.4458
1304.1674   1828.4142
1401.1525   1780.741
1475.1886   1237.7687
1806.191   2301.491
1947.2452   1572.4076
1948.1266   2861.5203
END IONS
BEGIN IONS
PEPMASS=2021.9513
CHARGE=1+
TITLE=Label: B7, Spot_Id: 3106644, Peak_List_Id: 6375349, MSMS Job_Run_Id: 57745, Comment:
174.93726   486.5098
900.2641   1878.5422
1835.2261   2253.5442
1837.0923   404.06787
1857.2061   470.22409
1975.2869   697.35101
1977.162   934.25714
END IONS
BEGIN IONS
PEPMASS=2043.0476
CHARGE=1+
TITLE=Label: B7, Spot_Id: 3106644, Peak_List_Id: 6375351, MSMS Job_Run_Id: 57745, Comment:
174.95258   435.9216
1033.1577   1108.7946
1034.3572   423.57559
1975.3502   276.66693
1991.2622   1146.4929
1995.1633   311.69135
END IONS
BEGIN IONS
PEPMASS=2070.0222
CHARGE=1+
TITLE=Label: B7, Spot_Id: 3106644, Peak_List_Id: 6375354, MSMS Job_Run_Id: 57745, Comment:

```
174.93808   326.58826
1033.1544   880.22449
1034.3684   461.26208
1394.1324   802.87695
1458.1107   1511.8247
1474.0581   209.19858
2004.2635   4491.1055
2005.2059   5902.4858
2008.0627   394.08774
2009.8821   633.64929
2010.7838   2125.718
2012.9006   611.32538
END IONS
BEGIN IONS
PEPMASS=2087.9597
CHARGE=1+
TITLE=Label: B7, Spot_Id: 3106644, Peak_List_Id: 63
75364, MSMS Job_Run_Id: 57745, Comment:
174.94817   3076.2344
288.01324   604.74823
313.94128   544.4151
397.94348   834.90088
457.02496   624.6698
474.04739   1050.688
497.00662   1275.2643
610.02728   565.98334
637.08221   1923.4058
734.047 1035.8141
739.02667   477.71494
751.06421   13744.848
752.17047   6659.4189
849.02588   555.81012
866.0611    860.01221
965.10529   540.84155
1036.1052   555.07831
1164.1432   962.68616
1261.0909   699.79187
1349.1338   2273.345
END IONS
BEGIN IONS
PEPMASS=2101.9629
CHARGE=1+
```

```
TITLE=Label: B7, Spot_Id: 3106644, Peak_List_Id: 63
75352, MSMS Job_Run_Id: 57745, Comment:
174.93741   918.63007
288.00418   200.86275
496.98758   212.98938
637.07642   321.43668
751.10394   2415.3091
767.27167   427.2984
2032.3059   508.30417
2035.1371   244.5289
END IONS
BEGIN IONS
PEPMASS=2150.1257
CHARGE=1+
TITLE=Label: B7, Spot_Id: 3106644, Peak_List_Id: 63
75361, MSMS Job_Run_Id: 57745, Comment:
174.94582   1586.9805
492.05716   23388.383
509.05258   471.64731
590.07318   681.98761
596.07843   843.55859
624.08936   513.5213
687.04614   1184.1876
704.06335   1557.4968
709.20337   659.75488
774.06091   676.72168
791.13214   1037.9695
808.1911    750.4845
970.09387   1058.2279
987.10907   1386.9796
988.28455   627.54236
1058.1675   1249.3131
1059.2803   544.35248
1155.1232   824.77856
1172.1373   492.0416
1243.174    491.43201
END IONS
BEGIN IONS
PEPMASS=2199.0493
CHARGE=1+
TITLE=Label: B7, Spot_Id: 3106644, Peak_List_Id: 63
75348, MSMS Job_Run_Id: 57745, Comment:
```

174.93541   226.31877
466.01981   1825.0093
492.06122   381.10742
651.1178   230.84229
765.10474   224.3667
904.14832   355.55551
1006.2142   316.18881
1548.2324   962.55914
1549.8466   271.73624
1733.2485   529.5946
2100.2961   1069.9313
2108.9009   382.53839
2149.4941   813.68335
2154.2207   607.99274
2157.23 314.36722
2158.3621   1282.2521
END IONS
BEGIN IONS
PEPMASS=2470.0762
CHARGE=1+
TITLE=Label: B7, Spot_Id: 3106644, Peak_List_Id: 63
75358, MSMS Job_Run_Id: 57745, Comment:
418.03598   472.23529
538.04443   519.34833
555.08331   973.33435
718.14368   781.08148
887.18488   415.70862
904.18683   16958.922
906.31134   1207.9327
1002.1638   629.45959
1019.1801   706.28766
1120.1766   413.96011
1233.1401   388.83759
1334.1672   509.758
1734.2952   999.5835
1863.2278   418.89758
1960.2828   402.5025
1977.2655   4393.8271
2191.3118   3162.7932
2425.2356   2823.9363
2429.1194   341.03864
2429.7937   431.06876

```
END IONS
BEGIN IONS
PEPMASS=3214.6296
CHARGE=1+
TITLE=Label: B7, Spot_Id: 3106644, Peak_List_Id: 63
75359, MSMS Job_Run_Id: 57745, Comment:
456.10034   716.39252
593.15802   982.23633
706.24445   875.98761
803.24445   520.41028
820.29523   550.87823
921.26239   2035.9385
1050.3186   246.84927
1356.377    244.02109
1373.4099   647.24792
1486.4542   221.22301
1769.5458   1306.0621
1870.5529   305.63791
1998.6682   268.52017
2398.6877   1543.5685
3169.8425   582.52356
3170.7349   2157.9236
3174.3313   636.35242
END IONS
BEGIN IONS
PEPMASS=3247.3425
CHARGE=1+
TITLE=Label: B7, Spot_Id: 3106644, Peak_List_Id: 63
75353, MSMS Job_Run_Id: 57745, Comment:
174.96085   235.68628
623.1142    370.0209
720.15393   249.65102
794.23529   221.69208
2013.4584   457.83923
2113.4988   3798.1638
2233.5063   2038.7023
3084.4004   717.65033
END IONS
```
