# Supplementary material for: A novel multicopper oxidase (laccase) from cyanobacteria: Purification, characterization with potential in the decolorization of anthraquinonic dye
Source: PLoS One. 2017 Apr 6;12(4):e0175144. doi: 10.1371/journal.pone.0175144 (PMC5383238; doi:10.1371/journal.pone.0175144)
Supplement: S2 File — (PDF) [file pone.0175144.s002.pdf]

MATRIX SCIENCE MASCOT Search Results

Protein View: AMT85333.1

laccase [Trametes versicolor]

**Database:**	NCBIprot
**Score:**	311
**Expect:**	9.1e-24
**Monoisotopic mass (M_r):**	56098
**Calculated pI:**	5.98
**Taxonomy:**	Trametes versicolor

Sequence similarity is available as an NCBI BLAST search of AMT85333.1 against nr.

Search parameters

**MS data file:**	Supplementary Figure 1.txt
**Enzyme:**	Trypsin: cuts C-term side of KR unless next residue is P.
**Fixed modifications:**	Carbamidomethyl (C)

Protein sequence coverage: 24%

Matched peptides shown in ***bold red***.

1 MGLQRFSFFV TLALVAR**SLA AIGPVASLVV ANAPVSPDDF LRDAIVVNGV**
51 **VPSPLITGK**K GDRFQLNVVD TLTNHSMLKS TSIHWHGFFQ AGTNWADGPA
101 FVNQCPIASG HSFLYDFHVP DQAGTFWYHS RLSTQYCDGL RGPFVVYDPK
151 DPHASR**YDVD NESTVITLTD WYHTAAR**LGP R**FPLGADATL INGLGR**SAST
201 PTAALAVINV QHGKRYRLRL VSISCDPNYT FSIDGHNLTV IEVDGINSQP
251 LLVDSIQIFA AQRYSFVLNA NQTVGNYWVR **ANPNFGTVGF AGGINSAILR**
301 **YQGAPVAEPT TTQTPSVIPL IETNLHPLAR** MPVPGSPTPG GVDKALNLAF
351 NFNGTNFFIN NASFTPPTVP VLLQILSGAQ TAQELLPAGS VYPLPAHSTI
401 EITLPATALA PGAPHPFHLH GHAFAVVRSA GSTTYNYNDP IFRDVVSTGT
451 PAAGDNVTIR FQTDNPGPWF LHCHIDFHLE AGFAIVFAED VADVKAANPV
501 PKAWSDLCPI YDGLSEANQ

Unformatted sequence string: 519 residues (for pasting into other applications).

Sort by ◉ residue number ○ increasing mass ○ decreasing mass
Show ◉ matched peptides only ○ predicted peptides also

Query	Start - End	Observed	Mr(expt)	Mr(calc)	Delta	M	Score	Peptide
38	18 - 59	2070.0339	4138.0532	4138.3042	-0.2510	1		R.SLAAIGPVASLVVANAPVSPDDFLRDAIVVNGVVPSPLITGK.K
25	157 - 177	2470.0762	2469.0689	2469.1448	-0.0759	0	66	R.YDVDNESTVITLTDWYHTAAR.L
10	182 - 196	1514.7855	1513.7782	1513.8253	-0.0470	0	38	R.FPLGADATLINGLGR.S
16	281 - 300	1975.9897	1974.9824	1975.0276	-0.0451	0	92	R.ANPNFGTVGFAGGINSAILR.Y
30	301 - 330	3214.6296	3213.6223	3213.7034	-0.0810	0	90	R.YQGAPVAEPTTTQTPSVIPLIETNLHPLAR.M

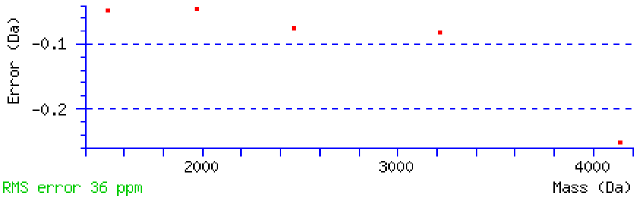

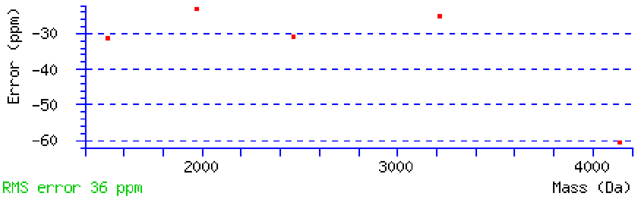


```
LOCUS       AMT85333                 519 aa            linear   PLN 05-APR-2016
DEFINITION  laccase [Trametes versicolor].
ACCESSION   AMT85333
VERSION     AMT85333.1
DBSOURCE    accession KR492189.1
KEYWORDS    .
SOURCE      Trametes versicolor (Coriolus versicolor)
  ORGANISM  Trametes versicolor
            Eukaryota; Fungi; Dikarya; Basidiomycota; Agaricomycotina;
            Agaricomycetes; Polyporales; Trametes.
REFERENCE   1  (residues 1 to 519)
  AUTHORS   Bertrand,B., Martinez-Morales,F., Tinoco-Valencia,R., Rojas,S.,
            Acosta-Urdapilleta,L. and Trejo-Hernandez,M.D.R.
  TITLE     Biochemical and molecular characterization of laccase isoforms
            produced by the white-rot fungus Trametes versicolor under
            submerged culture conditions
  JOURNAL   J. Mol. Catal., B Enzym. 122, 339-347 (2015)
REFERENCE   2  (residues 1 to 519)
  AUTHORS   Bertrand,B., Martinez-Morales,F. and Trejo-Hernandez,M.D.R.
  TITLE     Direct Submission
  JOURNAL   Submitted (27-APR-2015) Environmental Biotechnology, Centro de
            Investigacion en Biotecnologia (CEIB-UAEM), Av. Universidad No.
```

```
            1001, Col Chamilpa, Cuernavaca, Morelos 62209, Mexico
COMMENT     ##Assembly-Data-START##
            Assembly Method       :: BioEdit v. 7.0.5.3
            Sequencing Technology :: Illumina
            ##Assembly-Data-END##
FEATURES             Location/Qualifiers
     source          1..519
                     /organism="Trametes versicolor"
                     /isolate="K4"
                     /db_xref="taxon:5325"
     Protein         1..519
                     /product="laccase"
                     /EC_number="1.10.3.2"
     CDS             1..519
                     /coded_by="KR492189.1:1..1560"
```

Mascot: http://www.matrixscience.com/
