# Supplementary material for: A novel multicopper oxidase (laccase) from cyanobacteria: Purification, characterization with potential in the decolorization of anthraquinonic dye
Source: PLoS One. 2017 Apr 6;12(4):e0175144. doi: 10.1371/journal.pone.0175144 (PMC5383238; doi:10.1371/journal.pone.0175144)
Supplement: S3 File — (PDF) [file pone.0175144.s003.pdf]

MATRIX SCIENCE Mascot Search Results

User	:	Sumbul
Email	:	sumbulafreen12@gmail.com
Search title	:	Project: ABSCIEX SERVICE, Spot Set: ABSCIEX SERVICE\080316, Label: B7, Spot Id: 3106644, Peak List Id: 6375246, MS ‹
MS data file	:	Supplementary Figure 1.txt
Database 1	:	NCBIprot 20170215 (114247375 sequences; 41883446350 residues)
Database 2	:	SwissProt 2017_02 (553655 sequences; 198177566 residues)
Timestamp	:	22 Feb 2017 at 11:48:21 GMT
Warning	:	A Peptide summary report will usually give a much clearer picture of MS/MS search results.
Top Score	:	311 for 1::AMT85333.1, laccase [Trametes versicolor]

Mascot Score Histogram

Protein score is -10*Log(P), where P is the probability that the observed match is a random event.
Protein scores greater than 93 are significant (p<0.05).
Protein scores are derived from ions scores as a non-probabilistic basis for ranking protein hits.

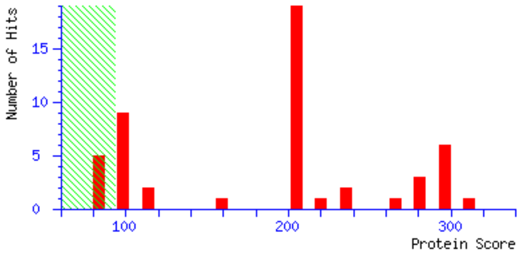

Protein Summary Report

Format As | Protein Summary (deprecated) | Help | Help

Significance threshold p< 0.05 | Max. number of hits 20

Preferred taxonomy All entries

Re-Search All | Search Unmatched

Index

	Accession	Mass	Score	Description
1.	1::AMT85333.1	56098	311	laccase [Trametes versicolor]
2.	1::AMT85332.1	50395	300	laccase [Trametes versicolor]
3.	2::LAC2_TRAVI	56027	299	Laccase-2 OS=Trametes villosa GN=LCC2 PE=3 SV=1
4.	1::Q99046.1	56027	299	RecName: Full=Laccase-2; AltName: Full=Benzenediol:oxygen oxidoreductase 2; AltName: Full=Diphenol o›
5.	1::AAW29420.1	56056	299	laccase 1 [Trametes versicolor]
6.	1::AMT85329.1	56083	299	laccase [Trametes versicolor]
7.	1::AMT85330.1	56055	299	laccase [Trametes versicolor]
8.	1::CAA77015.1	55740	287	laccase [Trametes versicolor]
9.	1::AAL93622.1	55724	287	laccase III [Trametes versicolor]
10.	1::ADK55593.1	55824	287	laccase [Trametes sp. 48424]
11.	1::AAL00887.1	56248	267	laccase 1 [Trametes versicolor]
12.	1::AMT85331.1	55925	229	laccase [Trametes versicolor]
13.	1::BAD98306.1	55992	229	laccase2 [Trametes versicolor]
14.	1::AIZ72725.1	56499	218	laccase F [Trametes hirsuta]
15.	1::AAM18407.1	55770	212	laccase 2 [Trametes pubescens]
16.	1::ACG61163.1	43144	211	laccase, partial [Trametes versicolor]
17.	1::ADE44157.1	56241	205	laccase [Trametes velutina]
18.	1::1GYC_A	53885	202	Chain A, Crystal Structure Determination At Room Temperature Of A Laccase From Trametes Versicolor Ir
19.	2::LAC2_TRAVE	56061	202	Laccase-2 OS=Trametes versicolor GN=LCC2 PE=1 SV=1
20.	1::AFM31222.1	56033	202	laccase [Trametes versicolor]

Results List

1. 1::AMT85333.1 **Mass:** 56098 **Score:** 311 **Expect:** 9.1e-24 **Matches:** 5

laccase [Trametes versicolor]

Observed	Mr(expt)	Mr(calc)	Delta	Start		End	Miss	Ions	Peptide
1514.7855	1513.7782	1513.8253	-0.0470	182	-	196	0	38	R.FPLGADATLINGLGR.S
1975.9897	1974.9824	1975.0276	-0.0451	281	-	300	0	92	R.ANPNFGTVGFAGGINSAILR.Y
2470.0762	2469.0689	2469.1448	-0.0759	157	-	177	0	66	R.YDVDNESTVITLTDWYHTAAR.L
3214.6296	3213.6223	3213.7034	-0.0810	301	-	330	0	90	R.YQGAPVAEPTTTQTPSVIPLIETNLHPLAR.M
2070.0339	4138.0532	4138.3042	-0.2510	18	-	59	1	---	R.SLAAIGPVASLVVANAPVSPDDFLRDAIVVNGVVPSPLITGK.K

No match to: 731.4565, 745.4716, 751.3851, 761.4732, 804.2769, 819.5120, 832.3038, 1243.6450, 1474.6757, 832.3132, 834.3159, 1703.7522, 1742.7133, 1948.0842, 1991.9727, 2021.9513, 2043.0476, 2070.0222, 2087.9597, 2101.9629, 2150.1257, 2199.0493, 1243.6581, 1474.6874, 1514.7983, 1515.7916, 3247.3425, 1703.7673, 1705.7672, 1974.0721, 1976.0077, 1991.9894, 2059.9446, 2087.9773, 2150.1462, 2158.0591, 2199.0723, 2470.0964, 3214.6680, 3247.3762

2. 1::AMT85332.1 **Mass:** 50395 **Score:** 300 **Expect:** 1.1e-22 **Matches:** 4

laccase [Trametes versicolor]

Observed	Mr(expt)	Mr(calc)	Delta	Start		End	Miss	Ions	Peptide
1514.7855	1513.7782	1513.8253	-0.0470	182	-	196	0	38	R.FPLGADATLINGLGR.S
1975.9897	1974.9824	1975.0276	-0.0451	281	-	300	0	92	R.ANPNFGTVGFAGGINSAILR.Y
2470.0762	2469.0689	2469.1448	-0.0759	157	-	177	0	66	R.YDVDNESTVITLTDWYHTAAR.L

3214.6296	3213.6223	3213.7034	-0.0810	301 - 330	0	90	R.YQGAPVAEPTTTQTPSVIPLIETNLHPLAR.M

No match to: 731.4565, 745.4716, 751.3851, 761.4732, 804.2769, 819.5120, 832.3038, 1243.6450, 1474.6757, 832.3132, 834.3159, 1703.7522, 1742.7133, 1948.0842, 1991.9727, 2021.9513, 2043.0476, 2070.0222, 2087.9597, 2101.9629, 2150.1257, 2199.0493, 1243.6581, 1474.6874, 1514.7983, 1515.7916, 3247.3425, 1703.7673, 1705.7672, 1974.0721, 1976.0077, 1991.9894, 2059.9446, 2070.0339, 2087.9773, 2150.1462, 2158.0591, 2199.0723, 2470.0964, 3214.6680, 3247.3762

3. 2::LAC2_TRAVI **Mass:** 56027 **Score:** 299 **Expect:** 1.4e-22 **Matches:** 4

Laccase-2 OS=Trametes villosa GN=LCC2 PE=3 SV=1

Observed	Mr(expt)	Mr(calc)	Delta	Start - End	Miss	Ions	Peptide
1514.7855	1513.7782	1513.8253	-0.0470	182 - 196	0	38	K.FPLGADATLINGLGR.S
1975.9897	1974.9824	1975.0276	-0.0451	281 - 300	0	92	R.ANPNFGTVGFAGGINSAILR.Y
2470.0762	2469.0689	2469.1448	-0.0759	157 - 177	0	66	R.YDVDNESTVITLTDWYHTAAR.L
3214.6296	3213.6223	3213.7034	-0.0810	301 - 330	0	90	R.YQGAPVAEPTTTQTPSVIPLIETNLHPLAR.M

No match to: 731.4565, 745.4716, 751.3851, 761.4732, 804.2769, 819.5120, 832.3038, 1243.6450, 1474.6757, 832.3132, 834.3159, 1703.7522, 1742.7133, 1948.0842, 1991.9727, 2021.9513, 2043.0476, 2070.0222, 2087.9597, 2101.9629, 2150.1257, 2199.0493, 1243.6581, 1474.6874, 1514.7983, 1515.7916, 3247.3425, 1703.7673, 1705.7672, 1974.0721, 1976.0077, 1991.9894, 2059.9446, 2070.0339, 2087.9773, 2150.1462, 2158.0591, 2199.0723, 2470.0964, 3214.6680, 3247.3762

4. 1::Q99046.1 **Mass:** 56027 **Score:** 299 **Expect:** 1.4e-22 **Matches:** 4

RecName: Full=Laccase-2; AltName: Full=Benzenediol:oxygen oxidoreductase 2; AltName: Full=Diphenol oxidase 2; AltName: Full=Uri

Observed	Mr(expt)	Mr(calc)	Delta	Start - End	Miss	Ions	Peptide
1514.7855	1513.7782	1513.8253	-0.0470	182 - 196	0	38	K.FPLGADATLINGLGR.S
1975.9897	1974.9824	1975.0276	-0.0451	281 - 300	0	92	R.ANPNFGTVGFAGGINSAILR.Y
2470.0762	2469.0689	2469.1448	-0.0759	157 - 177	0	66	R.YDVDNESTVITLTDWYHTAAR.L
3214.6296	3213.6223	3213.7034	-0.0810	301 - 330	0	90	R.YQGAPVAEPTTTQTPSVIPLIETNLHPLAR.M

No match to: 731.4565, 745.4716, 751.3851, 761.4732, 804.2769, 819.5120, 832.3038, 1243.6450, 1474.6757, 832.3132, 834.3159, 1703.7522, 1742.7133, 1948.0842, 1991.9727, 2021.9513, 2043.0476, 2070.0222, 2087.9597, 2101.9629, 2150.1257, 2199.0493, 1243.6581, 1474.6874, 1514.7983, 1515.7916, 3247.3425, 1703.7673, 1705.7672, 1974.0721, 1976.0077, 1991.9894, 2059.9446, 2070.0339, 2087.9773, 2150.1462, 2158.0591, 2199.0723, 2470.0964, 3214.6680, 3247.3762

5. 1::AAW29420.1 **Mass:** 56056 **Score:** 299 **Expect:** 1.4e-22 **Matches:** 4

laccase 1 [Trametes versicolor]

Observed	Mr(expt)	Mr(calc)	Delta	Start - End	Miss	Ions	Peptide
1514.7855	1513.7782	1513.8253	-0.0470	182 - 196	0	38	R.FPLGADATLINGLGR.S
1975.9897	1974.9824	1975.0276	-0.0451	281 - 300	0	92	R.ANPNFGTVGFAGGINSAILR.Y
2470.0762	2469.0689	2469.1448	-0.0759	157 - 177	0	66	R.YDVDNESTVITLTDWYHTAAR.L
3214.6296	3213.6223	3213.7034	-0.0810	301 - 330	0	90	R.YQGAPVAEPTTTQTPSVIPLIETNLHPLAR.M

No match to: 731.4565, 745.4716, 751.3851, 761.4732, 804.2769, 819.5120, 832.3038, 1243.6450, 1474.6757, 832.3132, 834.3159, 1703.7522, 1742.7133, 1948.0842, 1991.9727, 2021.9513, 2043.0476, 2070.0222, 2087.9597, 2101.9629, 2150.1257, 2199.0493, 1243.6581, 1474.6874, 1514.7983, 1515.7916, 3247.3425, 1703.7673, 1705.7672, 1974.0721, 1976.0077, 1991.9894, 2059.9446, 2070.0339, 2087.9773, 2150.1462, 2158.0591, 2199.0723, 2470.0964, 3214.6680, 3247.3762

6. 1::AMT85329.1 **Mass:** 56083 **Score:** 299 **Expect:** 1.4e-22 **Matches:** 4

laccase [Trametes versicolor]

Observed	Mr(expt)	Mr(calc)	Delta	Start - End	Miss	Ions	Peptide
1514.7855	1513.7782	1513.8253	-0.0470	182 - 196	0	38	R.FPLGADATLINGLGR.S
1975.9897	1974.9824	1975.0276	-0.0451	281 - 300	0	92	R.ANPNFGTVGFAGGINSAILR.Y
2470.0762	2469.0689	2469.1448	-0.0759	157 - 177	0	66	R.YDVDNESTVITLTDWYHTAAR.L
3214.6296	3213.6223	3213.7034	-0.0810	301 - 330	0	90	R.YQGAPVAEPTTTQTPSVIPLIETNLHPLAR.M

No match to: 731.4565, 745.4716, 751.3851, 761.4732, 804.2769, 819.5120, 832.3038, 1243.6450, 1474.6757, 832.3132, 834.3159, 1703.7522, 1742.7133, 1948.0842, 1991.9727, 2021.9513, 2043.0476, 2070.0222, 2087.9597, 2101.9629, 2150.1257, 2199.0493, 1243.6581, 1474.6874, 1514.7983, 1515.7916, 3247.3425, 1703.7673, 1705.7672, 1974.0721, 1976.0077, 1991.9894, 2059.9446, 2070.0339, 2087.9773, 2150.1462, 2158.0591, 2199.0723, 2470.0964, 3214.6680, 3247.3762

7. 1::AMT85330.1 **Mass:** 56055 **Score:** 299 **Expect:** 1.4e-22 **Matches:** 4

laccase [Trametes versicolor]

Observed	Mr(expt)	Mr(calc)	Delta	Start - End	Miss	Ions	Peptide
1514.7855	1513.7782	1513.8253	-0.0470	182 - 196	0	38	R.FPLGADATLINGLGR.S
1975.9897	1974.9824	1975.0276	-0.0451	281 - 300	0	92	R.ANPNFGTVGFAGGINSAILR.Y
2470.0762	2469.0689	2469.1448	-0.0759	157 - 177	0	66	R.YDVDNESTVITLTDWYHTAAR.L
3214.6296	3213.6223	3213.7034	-0.0810	301 - 330	0	90	R.YQGAPVAEPTTTQTPSVIPLIETNLHPLAR.M

No match to: 731.4565, 745.4716, 751.3851, 761.4732, 804.2769, 819.5120, 832.3038, 1243.6450, 1474.6757, 832.3132, 834.3159, 1703.7522, 1742.7133, 1948.0842, 1991.9727, 2021.9513, 2043.0476, 2070.0222, 2087.9597, 2101.9629, 2150.1257, 2199.0493, 1243.6581, 1474.6874, 1514.7983, 1515.7916, 3247.3425, 1703.7673, 1705.7672, 1974.0721, 1976.0077, 1991.9894, 2059.9446, 2070.0339, 2087.9773, 2150.1462, 2158.0591, 2199.0723, 2470.0964, 3214.6680, 3247.3762

8. 1::CAA77015.1 **Mass:** 55740 **Score:** 287 **Expect:** 2.3e-21 **Matches:** 5

laccase [Trametes versicolor]

Observed	Mr(expt)	Mr(calc)	Delta	Start - End	Miss	Ions	Peptide
1474.6757	1473.6684	1473.7153	-0.0469	81 - 92	0	44	K.STSIHWHGFFQK.G
1703.7522	1702.7449	1702.7951	-0.0502	430 - 444	0	52	R.SAGSTVYNYDNPIFR.D
1975.9897	1974.9824	1975.0123	-0.0299	445 - 463	1	1	R.DVVSTGTPAAGDNVTIRFR.T
1991.9727	1990.9654	1991.0225	-0.0571	282 - 301	0	84	R.ANPSFGNVGFTGGINSAILR.Y
2087.9597	2086.9524	2087.0112	-0.0588	265 - 281	0	98	R.YSFVLEANQAVDNYWIR.A

No match to: 731.4565, 745.4716, 751.3851, 761.4732, 804.2769, 819.5120, 832.3038, 1243.6450, 1514.7855, 832.3132, 834.3159, 1742.7133, 1948.0842, 2021.9513, 2043.0476, 2070.0222, 2101.9629, 2150.1257, 2199.0493, 2470.0762, 1243.6581, 1474.6874, 1514.7983, 1515.7916, 3214.6296, 3247.3425, 1703.7673, 1705.7672, 1974.0721, 1976.0077, 1991.9894, 2059.9446, 2070.0339, 2087.9773, 2150.1462, 2158.0591, 2199.0723, 2470.0964, 3214.6680, 3247.3762

9. 1::AAL93622.1 **Mass:** 55724 **Score:** 287 **Expect:** 2.3e-21 **Matches:** 5

laccase III [Trametes versicolor]

Observed	Mr(expt)	Mr(calc)	Delta	Start - End	Miss	Ions	Peptide
1474.6757	1473.6684	1473.7153	-0.0469	81 - 92	0	44	K.STSIHWHGFFQK.G
1703.7522	1702.7449	1702.7951	-0.0502	430 - 444	0	52	R.SAGSTVYNYDNPIFR.D

1975.9897	1974.9824	1975.0123	-0.0299	445 - 463	1	1	R.DVVSTGTPAAGDNVTIRFR.T
1991.9727	1990.9654	1991.0225	-0.0571	282 - 301	0	84	R.ANPSFGNVGFTGGINSAILR.Y
2087.9597	2086.9524	2087.0112	-0.0588	265 - 281	0	98	R.YSFVLEANQAVDNYWIR.A

No match to: 731.4565, 745.4716, 751.3851, 761.4732, 804.2769, 819.5120, 832.3038, 1243.6450, 1514.7855, 832.3132, 834.3159, 1742.7133, 1948.0842, 2021.9513, 2043.0476, 2070.0222, 2101.9629, 2150.1257, 2199.0493, 2470.0762, 1243.6581, 1474.6874, 1514.7983, 1515.7916, 3214.6296, 3247.3425, 1703.7673, 1705.7672, 1974.0721, 1976.0077, 1991.9894, 2059.9446, 2070.0339, 2087.9773, 2150.1462, 2158.0591, 2199.0723, 2470.0964, 3214.6680, 3247.3762

10. 1::ADK55593.1 **Mass:** 55824 **Score:** 287 **Expect:** 2.3e-21 **Matches:** 5

laccase [Trametes sp. 48424]

Observed	Mr(expt)	Mr(calc)	Delta	Start - End	Miss	Ions	Peptide
1474.6757	1473.6684	1473.7153	-0.0469	81 - 92	0	44	K.STSIHWHGFFQK.G
1703.7522	1702.7449	1702.7951	-0.0502	430 - 444	0	52	R.SAGSTVYNYDNPIFR.D
1975.9897	1974.9824	1975.0123	-0.0299	445 - 463	1	1	R.DVVSTGTPAAGDNVTIRFR.T
1991.9727	1990.9654	1991.0225	-0.0571	282 - 301	0	84	R.ANPSFGNVGFTGGINSAILR.Y
2087.9597	2086.9524	2087.0112	-0.0588	265 - 281	0	98	R.YSFVLEANQAVDNYWIR.A

No match to: 731.4565, 745.4716, 751.3851, 761.4732, 804.2769, 819.5120, 832.3038, 1243.6450, 1514.7855, 832.3132, 834.3159, 1742.7133, 1948.0842, 2021.9513, 2043.0476, 2070.0222, 2101.9629, 2150.1257, 2199.0493, 2470.0762, 1243.6581, 1474.6874, 1514.7983, 1515.7916, 3214.6296, 3247.3425, 1703.7673, 1705.7672, 1974.0721, 1976.0077, 1991.9894, 2059.9446, 2070.0339, 2087.9773, 2150.1462, 2158.0591, 2199.0723, 2470.0964, 3214.6680, 3247.3762

11. 1::AAL00887.1 **Mass:** 56248 **Score:** 267 **Expect:** 2.3e-19 **Matches:** 4

laccase 1 [Trametes versicolor]

Observed	Mr(expt)	Mr(calc)	Delta	Start - End	Miss	Ions	Peptide
1975.9897	1974.9824	1975.0276	-0.0451	281 - 300	0	92	R.ANPNFGTVGFAGGINSAILR.Y
2470.0762	2469.0689	2469.1448	-0.0759	157 - 177	0	66	R.YDVDNESTVITLTDWYHTAAR.L
3214.6296	3213.6223	3213.7034	-0.0810	301 - 330	0	90	R.YQGAPVAEPTTTQTPSVIPLIETNLHPLAR.M
3247.3425	3246.3352	3245.7521	0.5832	182 - 214	1	---	R.FPLGADATVINGLGRSASTPTAALAVINVQHGK.R

No match to: 731.4565, 745.4716, 751.3851, 761.4732, 804.2769, 819.5120, 832.3038, 1243.6450, 1474.6757, 1514.7855, 832.3132, 834.3159, 1703.7522, 1742.7133, 1948.0842, 1991.9727, 2021.9513, 2043.0476, 2070.0222, 2087.9597, 2101.9629, 2150.1257, 2199.0493, 1243.6581, 1474.6874, 1514.7983, 1515.7916, 1703.7673, 1705.7672, 1974.0721, 1976.0077, 1991.9894, 2059.9446, 2070.0339, 2087.9773, 2150.1462, 2158.0591, 2199.0723, 2470.0964, 3214.6680, 3247.3762

12. 1::AMT85331.1 **Mass:** 55925 **Score:** 229 **Expect:** 1.4e-15 **Matches:** 3

laccase [Trametes versicolor]

Observed	Mr(expt)	Mr(calc)	Delta	Start - End	Miss	Ions	Peptide
1514.7855	1513.7782	1513.8253	-0.0470	181 - 195	0	38	R.FPLGADATLINGLGR.S
1975.9897	1974.9824	1975.0276	-0.0451	280 - 299	0	92	R.ANPNFGTVGFAGGINSAILR.Y
3214.6296	3213.6223	3213.7034	-0.0810	300 - 329	0	90	R.YQGAPVAEPTTTQTPSVIPLIETNLHPLAR.M

No match to: 731.4565, 745.4716, 751.3851, 761.4732, 804.2769, 819.5120, 832.3038, 1243.6450, 1474.6757, 832.3132, 834.3159, 1703.7522, 1742.7133, 1948.0842, 1991.9727, 2021.9513, 2043.0476, 2070.0222, 2087.9597, 2101.9629, 2150.1257, 2199.0493, 2470.0762, 1243.6581, 1474.6874, 1514.7983, 1515.7916, 3247.3425, 1703.7673, 1705.7672, 1974.0721, 1976.0077, 1991.9894, 2059.9446, 2070.0339, 2087.9773, 2150.1462, 2158.0591, 2199.0723, 2470.0964, 3214.6680, 3247.3762

13. 1::BAD98306.1 **Mass:** 55992 **Score:** 229 **Expect:** 1.4e-15 **Matches:** 3

laccase2 [Trametes versicolor]

Observed	Mr(expt)	Mr(calc)	Delta	Start - End	Miss	Ions	Peptide
1514.7855	1513.7782	1513.8253	-0.0470	182 - 196	0	38	R.FPLGADATLINGLGR.S
1975.9897	1974.9824	1975.0276	-0.0451	281 - 300	0	92	R.ANPNFGTVGFAGGINSAILR.Y
3214.6296	3213.6223	3213.7034	-0.0810	301 - 330	0	90	R.YQGAPVAEPTTTQTPSVIPLIETNLHPLAR.M

No match to: 731.4565, 745.4716, 751.3851, 761.4732, 804.2769, 819.5120, 832.3038, 1243.6450, 1474.6757, 832.3132, 834.3159, 1703.7522, 1742.7133, 1948.0842, 1991.9727, 2021.9513, 2043.0476, 2070.0222, 2087.9597, 2101.9629, 2150.1257, 2199.0493, 2470.0762, 1243.6581, 1474.6874, 1514.7983, 1515.7916, 3247.3425, 1703.7673, 1705.7672, 1974.0721, 1976.0077, 1991.9894, 2059.9446, 2070.0339, 2087.9773, 2150.1462, 2158.0591, 2199.0723, 2470.0964, 3214.6680, 3247.3762

14. 1::AIZ72725.1 **Mass:** 56499 **Score:** 218 **Expect:** 1.8e-14 **Matches:** 3

laccase F [Trametes hirsuta]

Observed	Mr(expt)	Mr(calc)	Delta	Start - End	Miss	Ions	Peptide
1474.6757	1473.6684	1473.7153	-0.0469	85 - 96	0	44	K.STSIHWHGFFQK.G
1975.9897	1974.9824	1976.0480	-1.0655	286 - 305	0	73	R.ANPLFGTTGFAGGINSAILR.Y
2087.9597	2086.9524	2087.0112	-0.0588	269 - 285	0	98	R.YSFVLEANQAVDNYWIR.A

No match to: 731.4565, 745.4716, 751.3851, 761.4732, 804.2769, 819.5120, 832.3038, 1243.6450, 1514.7855, 832.3132, 834.3159, 1703.7522, 1742.7133, 1948.0842, 1991.9727, 2021.9513, 2043.0476, 2070.0222, 2101.9629, 2150.1257, 2199.0493, 2470.0762, 1243.6581, 1474.6874, 1514.7983, 1515.7916, 3214.6296, 3247.3425, 1703.7673, 1705.7672, 1974.0721, 1976.0077, 1991.9894, 2059.9446, 2070.0339, 2087.9773, 2150.1462, 2158.0591, 2199.0723, 2470.0964, 3214.6680, 3247.3762

15. 1::AAM18407.1 **Mass:** 55770 **Score:** 212 **Expect:** 7.2e-14 **Matches:** 5

laccase 2 [Trametes pubescens]

Observed	Mr(expt)	Mr(calc)	Delta	Start - End	Miss	Ions	Peptide
1474.6757	1473.6684	1473.7153	-0.0469	81 - 92	0	44	K.STSIHWHGFFQK.G
1703.7522	1702.7449	1702.7951	-0.0502	430 - 444	0	52	R.SAGSTVYNYDNPIFR.D
1975.9897	1974.9824	1975.0123	-0.0299	445 - 463	1	1	R.DVVSTGTPAAGDNVTIRFR.T
2087.9597	2086.9524	2087.0112	-0.0588	265 - 281	0	98	R.YSFVLEANQAVDNYWIR.A
2158.0591	4314.1036	4313.2631	0.8406	1 - 43	1	---	-.MSRFQSLLAFVVASLAAVAHAGIGPVADLTISNAAVSPDGFSR.Q

No match to: 731.4565, 745.4716, 751.3851, 761.4732, 804.2769, 819.5120, 832.3038, 1243.6450, 1514.7855, 832.3132, 834.3159, 1742.7133, 1948.0842, 1991.9727, 2021.9513, 2043.0476, 2070.0222, 2101.9629, 2150.1257, 2199.0493, 2470.0762, 1243.6581, 1474.6874, 1514.7983, 1515.7916, 3214.6296, 3247.3425, 1703.7673, 1705.7672, 1974.0721, 1976.0077, 1991.9894, 2059.9446, 2070.0339, 2087.9773, 2150.1462, 2199.0723, 2470.0964, 3214.6680, 3247.3762

16. 1::ACG61163.1 **Mass:** 43144 **Score:** 211 **Expect:** 9.1e-14 **Matches:** 4

laccase, partial [Trametes versicolor]

Observed	Mr(expt)	Mr(calc)	Delta	Start - End	Miss	Ions	Peptide
1514.7855	1513.7782	1513.8253	-0.0470	99 - 113	0	38	R.FPLGADATLINGLGR.S
1975.9897	1974.9824	1975.0276	-0.0451	198 - 217	0	92	R.ANPNFGTVGFAGGINSAILR.Y

2470.0762	2469.0689	2469.1448	-0.0759	74	-	94	0	66	R.YDVDNESTVITLTDWYHTAAR.L
3247.3425	3246.3352	3245.7408	0.5944	218	-	247	1	---	R.YRGAPVAEPTTTQTTSVIPLIETNLHPLAR.M

No match to: 731.4565, 745.4716, 751.3851, 761.4732, 804.2769, 819.5120, 832.3038, 1243.6450, 1474.6757, 832.3132, 834.3159, 1703.7522, 1742.7133, 1948.0842, 1991.9727, 2021.9513, 2043.0476, 2070.0222, 2087.9597, 2101.9629, 2150.1257, 2199.0493, 1243.6581, 1474.6874, 1514.7983, 1515.7916, 3214.6296, 1703.7673, 1705.7672, 1974.0721, 1976.0077, 1991.9894, 2059.9446, 2070.0339, 2087.9773, 2150.1462, 2158.0591, 2199.0723, 2470.0964, 3214.6680, 3247.3762

17. 1::ADE44157.1 **Mass:** 56241 **Score:** 205 **Expect:** 3.6e-13 **Matches:** 3

laccase [Trametes velutina]

Observed	Mr(expt)	Mr(calc)	Delta	Start		End	Miss	Ions	Peptide
1514.7855	1513.7782	1513.8253	-0.0470	183	-	197	0	38	K.FPLGADATLINGLGR.S
2470.0762	2469.0689	2469.1448	-0.0759	158	-	178	0	66	R.YDVDNESTVITLTDWYHTAAR.L
3214.6296	3213.6223	3213.7034	-0.0810	302	-	331	0	90	R.YQGAPVAEPTTTQTPSVIPLIETNLHPLAR.M

No match to: 731.4565, 745.4716, 751.3851, 761.4732, 804.2769, 819.5120, 832.3038, 1243.6450, 1474.6757, 832.3132, 834.3159, 1703.7522, 1742.7133, 1948.0842, 1975.9897, 1991.9727, 2021.9513, 2043.0476, 2070.0222, 2087.9597, 2101.9629, 2150.1257, 2199.0493, 1243.6581, 1474.6874, 1514.7983, 1515.7916, 3247.3425, 1703.7673, 1705.7672, 1974.0721, 1976.0077, 1991.9894, 2059.9446, 2070.0339, 2087.9773, 2150.1462, 2158.0591, 2199.0723, 2470.0964, 3214.6680, 3247.3762

18. 1::1GYC_A **Mass:** 53885 **Score:** 202 **Expect:** 7.2e-13 **Matches:** 3

Chain A, Crystal Structure Determination At Room Temperature Of A Laccase From Trametes Versicolor In Its Oxidised Form Contain

Observed	Mr(expt)	Mr(calc)	Delta	Start		End	Miss	Ions	Peptide
1514.7855	1513.7782	1513.8253	-0.0470	162	-	176	0	38	R.FPLGADATLINGLGR.S
1975.9897	1974.9824	1975.0276	-0.0451	261	-	280	0	92	R.ANPNFGTVGFAGGINSAILR.Y
2470.0762	2469.0689	2469.1448	-0.0759	137	-	157	0	66	R.YDVDNESTVITLTDWYHTAAR.L

No match to: 731.4565, 745.4716, 751.3851, 761.4732, 804.2769, 819.5120, 832.3038, 1243.6450, 1474.6757, 832.3132, 834.3159, 1703.7522, 1742.7133, 1948.0842, 1991.9727, 2021.9513, 2043.0476, 2070.0222, 2087.9597, 2101.9629, 2150.1257, 2199.0493, 1243.6581, 1474.6874, 1514.7983, 1515.7916, 3214.6296, 3247.3425, 1703.7673, 1705.7672, 1974.0721, 1976.0077, 1991.9894, 2059.9446, 2070.0339, 2087.9773, 2150.1462, 2158.0591, 2199.0723, 2470.0964, 3214.6680, 3247.3762

19. 2::LAC2_TRAVE **Mass:** 56061 **Score:** 202 **Expect:** 7.2e-13 **Matches:** 3

Laccase-2 OS=Trametes versicolor GN=LCC2 PE=1 SV=1

Observed	Mr(expt)	Mr(calc)	Delta	Start		End	Miss	Ions	Peptide
1514.7855	1513.7782	1513.8253	-0.0470	182	-	196	0	38	R.FPLGADATLINGLGR.S
1975.9897	1974.9824	1975.0276	-0.0451	281	-	300	0	92	R.ANPNFGTVGFAGGINSAILR.Y
2470.0762	2469.0689	2469.1448	-0.0759	157	-	177	0	66	R.YDVDNESTVITLTDWYHTAAR.L

No match to: 731.4565, 745.4716, 751.3851, 761.4732, 804.2769, 819.5120, 832.3038, 1243.6450, 1474.6757, 832.3132, 834.3159, 1703.7522, 1742.7133, 1948.0842, 1991.9727, 2021.9513, 2043.0476, 2070.0222, 2087.9597, 2101.9629, 2150.1257, 2199.0493, 1243.6581, 1474.6874, 1514.7983, 1515.7916, 3214.6296, 3247.3425, 1703.7673, 1705.7672, 1974.0721, 1976.0077, 1991.9894, 2059.9446, 2070.0339, 2087.9773, 2150.1462, 2158.0591, 2199.0723, 2470.0964, 3214.6680, 3247.3762

20. 1::AFM31222.1 **Mass:** 56033 **Score:** 202 **Expect:** 7.2e-13 **Matches:** 3

laccase [Trametes versicolor]

Observed	Mr(expt)	Mr(calc)	Delta	Start		End	Miss	Ions	Peptide
1514.7855	1513.7782	1513.8253	-0.0470	182	-	196	0	38	K.FPLGADATLINGLGR.S
1975.9897	1974.9824	1975.0276	-0.0451	281	-	300	0	92	R.ANPNFGTVGFAGGINSAILR.Y
2470.0762	2469.0689	2469.1448	-0.0759	157	-	177	0	66	R.YDVDNESTVITLTDWYHTAAR.L

No match to: 731.4565, 745.4716, 751.3851, 761.4732, 804.2769, 819.5120, 832.3038, 1243.6450, 1474.6757, 832.3132, 834.3159, 1703.7522, 1742.7133, 1948.0842, 1991.9727, 2021.9513, 2043.0476, 2070.0222, 2087.9597, 2101.9629, 2150.1257, 2199.0493, 1243.6581, 1474.6874, 1514.7983, 1515.7916, 3214.6296, 3247.3425, 1703.7673, 1705.7672, 1974.0721, 1976.0077, 1991.9894, 2059.9446, 2070.0339, 2087.9773, 2150.1462, 2158.0591, 2199.0723, 2470.0964, 3214.6680, 3247.3762

Search Parameters

```
Type of search         : MS/MS Ion Search
Enzyme                 : Trypsin
Fixed modifications    : Carbamidomethyl (C)
Mass values            : Monoisotopic
Protein Mass           : Unrestricted
Peptide Mass Tolerance : ± 1.2 Da
Fragment Mass Tolerance: ± 0.6 Da
Max Missed Cleavages   : 1
Instrument type        : MALDI-TOF-TOF
   Query1 (731.4565,1+): Label: B7, Spot_Id: 3106644, Peak_List_Id: 6375342, MSMS Job_Run_Id: 57745, Comment:
   Query2 (745.4716,1+): Label: B7, Spot_Id: 3106644, Peak_List_Id: 6375350, MSMS Job_Run_Id: 57745, Comment:
   Query3 (751.3851,1+): Label: B7, Spot_Id: 3106644, Peak_List_Id: 6375343, MSMS Job_Run_Id: 57745, Comment:
   Query4 (761.4732,1+): Label: B7, Spot_Id: 3106644, Peak_List_Id: 6375345, MSMS Job_Run_Id: 57745, Comment:
   Query5 (804.2769,1+): Label: B7, Spot_Id: 3106644, Peak_List_Id: 6375347, MSMS Job_Run_Id: 57745, Comment:
   Query6 (819.5120,1+): Label: B7, Spot_Id: 3106644, Peak_List_Id: 6375344, MSMS Job_Run_Id: 57745, Comment:
   Query7 (832.3038,1+): Label: B7, Spot_Id: 3106644, Peak_List_Id: 6375360, MSMS Job_Run_Id: 57745, Comment:
  Query8 (1243.6450,1+): Label: B7, Spot_Id: 3106644, Peak_List_Id: 6375355, MSMS Job_Run_Id: 57745, Comment:
  Query9 (1474.6757,1+): Label: B7, Spot_Id: 3106644, Peak_List_Id: 6375356, MSMS Job_Run_Id: 57745, Comment:
 Query10 (1514.7855,1+): Label: B7, Spot_Id: 3106644, Peak_List_Id: 6375357, MSMS Job_Run_Id: 57745, Comment:
  Query11 (832.3132,2+): <no title>
  Query12 (834.3159,2+): <no title>
 Query13 (1703.7522,1+): Label: B7, Spot_Id: 3106644, Peak_List_Id: 6375362, MSMS Job_Run_Id: 57745, Comment:
 Query14 (1742.7133,1+): Label: B7, Spot_Id: 3106644, Peak_List_Id: 6375341, MSMS Job_Run_Id: 57745, Comment:
 Query15 (1948.0842,1+): Label: B7, Spot_Id: 3106644, Peak_List_Id: 6375346, MSMS Job_Run_Id: 57745, Comment:
 Query16 (1975.9897,1+): Label: B7, Spot_Id: 3106644, Peak_List_Id: 6375363, MSMS Job_Run_Id: 57745, Comment:
 Query17 (1991.9727,1+): Label: B7, Spot_Id: 3106644, Peak_List_Id: 6375365, MSMS Job_Run_Id: 57745, Comment:
 Query18 (2021.9513,1+): Label: B7, Spot_Id: 3106644, Peak_List_Id: 6375349, MSMS Job_Run_Id: 57745, Comment:
 Query19 (2043.0476,1+): Label: B7, Spot_Id: 3106644, Peak_List_Id: 6375351, MSMS Job_Run_Id: 57745, Comment:
 Query20 (2070.0222,1+): Label: B7, Spot_Id: 3106644, Peak_List_Id: 6375354, MSMS Job_Run_Id: 57745, Comment:
 Query21 (2087.9597,1+): Label: B7, Spot_Id: 3106644, Peak_List_Id: 6375364, MSMS Job_Run_Id: 57745, Comment:
 Query22 (2101.9629,1+): Label: B7, Spot_Id: 3106644, Peak_List_Id: 6375352, MSMS Job_Run_Id: 57745, Comment:
 Query23 (2150.1257,1+): Label: B7, Spot_Id: 3106644, Peak_List_Id: 6375361, MSMS Job_Run_Id: 57745, Comment:
 Query24 (2199.0493,1+): Label: B7, Spot_Id: 3106644, Peak_List_Id: 6375348, MSMS Job_Run_Id: 57745, Comment:
 Query25 (2470.0762,1+): Label: B7, Spot_Id: 3106644, Peak_List_Id: 6375358, MSMS Job_Run_Id: 57745, Comment:
 Query26 (1243.6581,2+): <no title>
```

```
Query27 (1474.6874,2+): <no title>
Query28 (1514.7983,2+): <no title>
Query29 (1515.7916,2+): <no title>
Query30 (3214.6296,1+): Label: B7, Spot_Id: 3106644, Peak_List_Id: 6375359, MSMS Job_Run_Id: 57745, Comment:
Query31 (3247.3425,1+): Label: B7, Spot_Id: 3106644, Peak_List_Id: 6375353, MSMS Job_Run_Id: 57745, Comment:
Query32 (1703.7673,2+): <no title>
Query33 (1705.7672,2+): <no title>
Query34 (1974.0721,2+): <no title>
Query35 (1976.0077,2+): <no title>
Query36 (1991.9894,2+): <no title>
Query37 (2059.9446,2+): <no title>
Query38 (2070.0339,2+): <no title>
Query39 (2087.9773,2+): <no title>
Query40 (2150.1462,2+): <no title>
Query41 (2158.0591,2+): <no title>
Query42 (2199.0723,2+): <no title>
Query43 (2470.0964,2+): <no title>
Query44 (3214.6680,2+): <no title>
Query45 (3247.3762,2+): <no title>
```

Mascot: http://www.matrixscience.com/
